# Abnormalities of the endocannabinoid system produce piercing nuclear hernias in migrating cerebral neurons

**DOI:** 10.1016/j.isci.2025.113188

**Published:** 2025-07-23

**Authors:** Yury M. Morozov, Pasko Rakic

**Affiliations:** 1 Department of Neuroscience, Yale University School of Medicine and Kavli Institute for Neuroscience, New Haven, CT 06510, USA

**Keywords:** Biological sciences, Cellular neuroscience, Developmental neuroscience, Natural sciences, Neuroscience

## Abstract

We are reporting powerful streams of chromatin rupturing the nuclear envelope (NE) and the plasma membrane of migrating cerebral neurons in mouse embryos, which we suggest naming “piercing nuclear hernia” (PNH). About 40% of migrating neurons in cannabinoid type 1 receptor knock-out (CB_1_R^−/−^) mouse embryos and in wildtype embryos exposed to CB_1_R agonists show NE rupture and/or PNH. This indicates that deviations from optimal functioning of the endocannabinoid system in under- or over-activity may trigger analogous mechanisms increasing intranuclear pressure and chromatin herniation. The cells from CB_1_R^−/−^ embryos showed pronounced ultrastructural disorders, such as high volume of herniated chromatin, mitochondrial fission, and negative correlation of the mitochondrial length with the volume of herniated chromatin. Catastrophic rupture of the nuclear and plasma membranes may provoke accidental cell death. At the same time, a fraction of neurons with PNH showed generally normal ultrastructure, which could indicate a mechanism of cell body repair.

## Highlights

• Neurons migrating through mouse embryo cerebral cortex show chromatin herniations
• Chromatin streams herniating from the nucleus may pierce the plasma membrane
• Piercing nuclear hernia is a form of ultrastructural cell pathology
• CB_1_ receptor knock-out or stimulation increases nuclear hernia volume and frequency

## Graphical abstract

**Figure:**
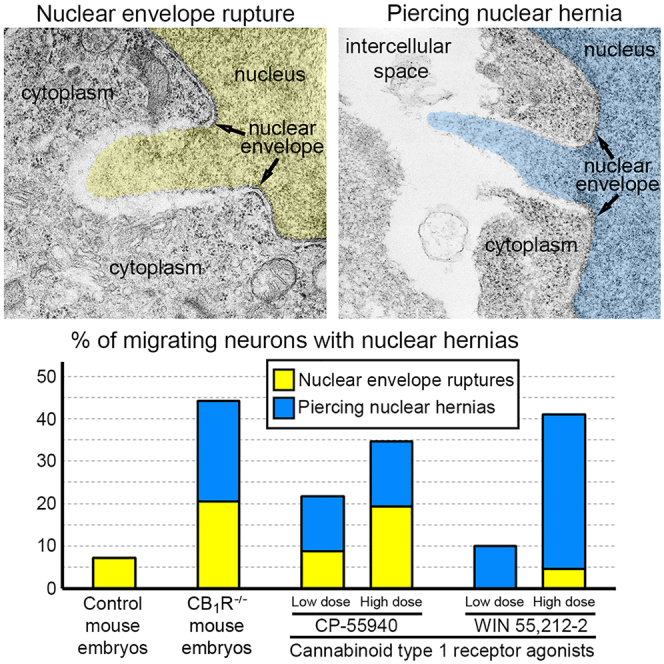

## Introduction

The endocannabinoid system has been identified in numerous phylogenetically distant animal clades, indicating it as an evolutionarily conserved trait.^1^^,^^2^^,^^3^^,^^4^^,^^5^^,^^6^ It is involved in several cognitive and physiological processes, including cardiovascular regulation, fertility, pregnancy, prenatal and postnatal development, activity of the immune system, food consumption, and energy metabolism.^7^^,^^8^^,^^9^^,^^10^^,^^11^^,^^12^^,^^13^^,^^14^^,^^15^ Endocannabinoid signaling via cannabinoid type 1 receptor (CB_1_R) often participates in retrograde synaptic modulation but also involves non-synaptic pathways.^16^^,^^17^^,^^18^ In addition to abundant expression in the axonal plasma membrane, CB_1_R accumulates in the membranes of intracellular vesicles in the cell bodies of certain types of adult and immature neurons.^19^^,^^20^^,^^21^ One demonstrated role of CB_1_R, which putatively involves the intracellular vesicles, is self-inhibition of cholecystokinin-expressing interneurons and a subpopulation of cortical pyramidal neurons through the cell’s autonomous Ca^2+^-dependent production of endocannabinoids and K^+^ channel activation.^22^^,^^23^ The endocannabinoid system influences synapse target selection by pyramidal neurons and GABAergic interneurons through CB_1_R internalization from axonal filopodia and chemorepulsion of the growth cones.^24^^,^^25^ We recently detected initial expression of CB_1_R in immature projection neurons vertically migrating through the cerebral intermediate zone and cortical plate, as well as in interneurons tangentially migrating through the marginal zone. We also demonstrated that, in CB_1_R^−/−^ embryos, migrating cerebral projection neurons emitted an increased number of processes and deviated from the vertical orientation, implicating CB_1_R’s role in cellular migration.^21^ However, molecular mechanisms of the endocannabinoid system in the cytoarchitecture of the developing brain are still enigmatic.

Therapeutic benefits of cannabis as an analgesic and antiemetic agent are well-known and applied for centuries.^26^^,^^27^ Inhibition of MAGL (monoacylglycerol lipase – enzyme degrading endocannabinoid 2-arachidonyl glycerol) has been proposed as a potential therapeutic approach for treatment of diverse neurological and neurodegenerative diseases, such as multiple sclerosis, Alzheimer’s disease, Parkinson’s disease, amyotrophic lateral sclerosis, and traumatic brain injury.^28^^,^^29^^,^^30^^,^^31^^,^^32^^,^^33^^,^^34^ Cannabinoid-based anti-epileptic and anti-spasticity medications such as Epidiolex and nabiximols (Sativex) have demonstrated efficacy in randomized controlled trials.^35^^,^^36^ Two cannabinoid drugs (dronabinol and nabilone) are approved by the US Food and Drug Administration for the prevention or treatment of cancer-related side effects, such as nausea and vomiting. *In vitro* and *in vivo* experiments on cancer models show that cannabinoids can kill cancer cells, making cannabis-based medicine a promising option to effectively modulate growth of certain tumor types. However, suspected anticancer benefits of cannabinoids have yet to be confirmed in clinical trials.^37^^,^^38^^,^^39^^,^^40^^,^^41^^,^^42^^,^^43^ Medical cannabis products are approved or tolerated in many countries and, increasingly, self-administered by pregnant individuals seeking relief from pain, nausea, or depression. The use of marijuana and synthetic cannabinoids during pregnancy and lactation poses potential risks for intrauterine and postnatal brain development, particularly as cannabinoids can appear in breast milk. In the absence of exhaustive biomedical studies, the extent to which marijuana and medicinal cannabinoids affect immature brains is still the subject of debate [Reviewed in^14^^,^^26^^,^^44^^,^^45^].

The nuclear envelope (NE) consists of the inner and outer nuclear membranes (INM and ONM, respectively) and the nuclear lamina – a composite matrix assembled on the inner nuclear membrane. The main function of the NE is to allow molecular exchange between the nucleoplasm and the cytoplasm through specified nuclear pores. Recent works highlighted the dynamic properties of the nuclear membranes and demonstrated that dysregulation of their functions has significant consequences for the cell.^46^^,^^47^^,^^48^^,^^49^^,^^50^ In addition to biochemical inputs, nuclei of the cells may be exposed to intrinsic and extrinsic mechanical forces transmitted by the cytoskeleton and nucleoskeleton that trigger dynamic changes in nuclear morphology.^46^^,^^48^^,^^49^^,^^51^ Cytoskeleton components (such as centrosome, microtubules, actin, myosin II, dynein, etc.) fulfill a crucial role in nucleus translocation, as demonstrated in migrating neurons.^52^^,^^53^^,^^54^^,^^55^ Emerging data suggests that extensive deformation of the cell and its nucleus during constrained migration transmits substantial physical stress on the NE and may result in its rupture, which in turn leads to chromatin herniation, uncontrolled exchange of nucleo-cytoplasmic content, DNA damage, and cell death.^46^^,^^56^^,^^57^^,^^58^^,^^59^ Distribution of a diffusible fluorescent marker from ruptured nucleus through the cytoplasm was observed as early as minutes after NE rupture, indicating the high speed of the reaction.^57^^,^^60^^,^^61^^,^^62^^,^^63^ The full impact of nucleo-cytoplasmic mixing is likely to be extensive yet poorly understood. Ruptures of the NE have been observed in several species *in vitro* and *in vivo* indicating that it is a consequence of the environmental conditions rather than a species-specific phenomenon [Reviewed in^64^]. For example, NE rupture was observed in neurons migrating during brain development.^56^^,^^65^ Further, NE ruptures were extensively studied in many cancer cell lines during cell migration through tightly constricted areas.^47^^,^^57^^,^^58^^,^^60^ NE ruptures can be induced in non-tumorigenic cell lines by generating a lamin B1 deficiency or depletion of two major tumor suppressors, p53 and retinoblastoma (Rb1).^56^^,^^66^^,^^67^ Cultivated cells also can be provoked to experience NE rupture by external mechanical force,^51^^,^^58^^,^^68^ human immunodeficiency virus infection,^62^ and activation of apoptosis, inflammation, or autophagy.^66^^,^^69^^,^^70^^,^^71^ Depletion, or mutation of nuclear lamin proteins as well as conditions that impair the connections between the INM and the chromatin increase the probability of NE ruptures.^47^^,^^48^^,^^56^^,^^57^^,^^59^^,^^60^^,^^68^^,^^72^^,^^73^^,^^74^^,^^75^ Thus, NE ruptures may be caused by defects in lamina organization, or increased intranuclear pressure transmitted by actin cytoskeleton and/or nucleoskeleton.^72^ Several NE repair mechanisms have been proposed, including attachment of endoplasmic reticulum sheets to the exposed chromatin, spreading of the preserved ONM, plugging using membrane fragments, and resealing by protein complexes [Reviewed in^64^^,^^76^]. Migrating cells reseal their ruptured NE using ESCRT-III (the endosomal sorting complexes required for transport III) and by doing so reduce DNA damage and cell death.^57^^,^^58^ An individual NE rupture may persist for minutes to hours before repair, however unrepaired NE ruptures eventually lead the cells to death.^57^^,^^58^^,^^60^^,^^61^^,^^77^

Here, extending our previous study of the role of endocannabinoid signaling in the developing brain,^20^^,^^21^^,^^24^^,^^78^^,^^79^ we performed extensive electron microscopy analysis with 3D reconstruction of migrating neurons in CB_1_R^−/−^ embryos, as well as after *in utero* application of CB_1_R agonists in wildtype mice. Morpho-functional conditions of mitochondria correlate with general cellular functionality, while swelling of the mitochondrial matrix and predominance of fission over fusion may serve as evidence of disordered cellular energetics or other malfunctions.^80^^,^^81^^,^^82^ This inspired our choice of ultrastructural and morphometric analyses of mitochondria as an indicator for potential cell disorder. We unexpectedly discovered a correlation between the frequency of chromatin herniation and disorder of the endocannabinoid system, which reveals a possible cellular mechanism for known effects of cannabinoids in the developing brain.^14^ Our study of *in vivo* experimental conditions for increasing frequency and power of chromatin herniation may be instrumental for further investigation of the mechanisms of membrane rupture and repair, as well as in prospective medicinal applications of the obtained knowledge.

## Results

### Piercing nuclear hernia is an ultrastructural pathology of migrating neurons

**Figure 1:**
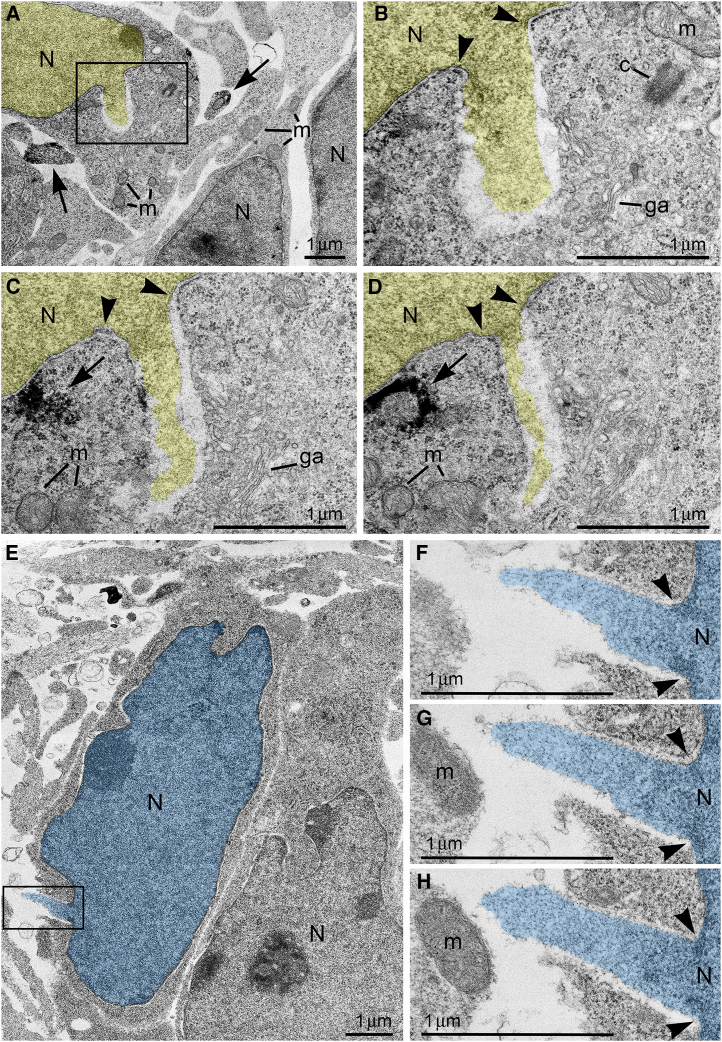
Ultrastructure of herniated chromatin streams (A–D) NE rupture in a neuron from the cortical plate of wildtype mouse embryo exposed to high dose (0.12 mg/kg) of CB1R agonist CP-55940. (A) Low power micrograph shows ultrastructure of neuronal cell bodies and neuropil that is generally normal for the developmental stage. (B–D) Serial high power images of NE rupture from the framed area in (A). Chromatin stream expelled from the nucleus (pseudo-colored yellow) is not surrounded by the nuclear membranes. Arrowheads indicate points of interruption of the nuclear membranes. Arrows point to CB1R-positive axons in (A) and CB1R-positive intracellular vesicle in (C) and (D). (E–H) Piercing nuclear hernia (PNH) in a neuron from the cortical plate of CB1R−/− embryo. (E) Low power micrograph shows generally normal ultrastructure of neurons. (F–H) Serial high power images of PNH from the framed area in (E). Note the continuum of the chromatin stream expelled from the nucleus (pseudo-colored blue) and penetrating the intercellular space. Arrowheads indicate points of interruption of the nuclear membranes. Abbreviations: c, centriole; ga, Golgi apparatus; m, mitochondria, N, nucleus.

**Figure 2:**
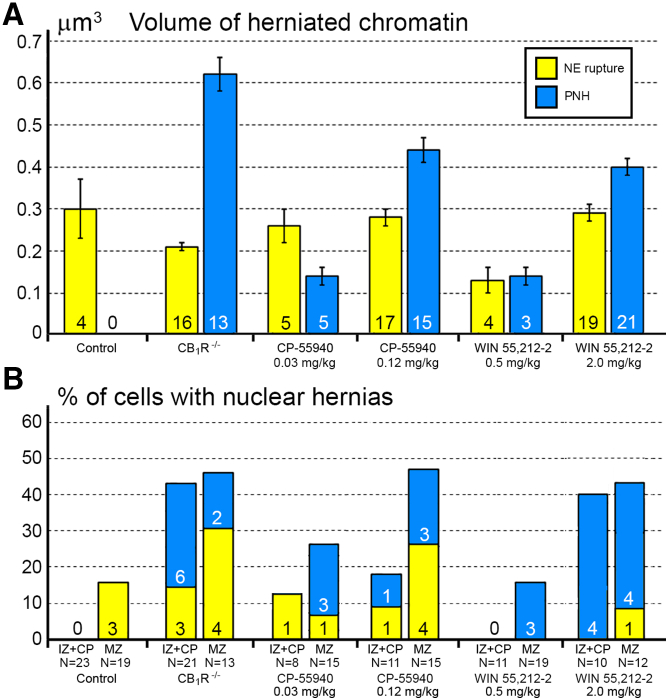
Volume and frequency of chromatin herniation through the experimental groups (A) Volume of herniated chromatin in cells from the embryo cerebrum of wildtype control mice, CB1R−/− mice and wildtype mice after CB1R agonists CP-55940 and WIN 55,212-2 applications at distinct doses. The data are represented in terms of average volume ±SEM of the 3D reconstructed streams of herniated chromatin. Numbers of analyzed NE ruptures and PNHs are indicated at the base of each column. Volumes of each analyzed hernia are shown in Table S1. (B) Percentages of the cells with nuclear hernias in the intermediate zone combined with the cortical plate (IZ + CP) and in the marginal zone (MZ) of the embryo cerebrum of wildtype control mice, CB1R−/− mice and wildtype mice exposed to CB1R agonists CP-55940 and WIN 55,212-2. Numbers of cells with NE ruptures and PNHs are indicated at the base of each column. N refers to the number of analyzed 3D reconstructed cells from corresponding groups. For complete morphologic characterizations of the 3D reconstructed cells see Figures 3, 4, and 5 and Tables S2, S3, S4, and S5.

Here, we report ultrastructural pathology in mouse embryo cerebral neurons related to CB_1_R knock-out and *in utero* application of synthetic cannabinoids. Particularly, we documented numerous local breakups of the NE accompanied by herniation of nuclear chromatin into the cytoplasm (referred as NE rupture hereafter; Figures 1A–1D ). Moreover, in a fraction of the cells, streams of herniated chromatin ruptured not only the NE, but also the plasma membrane, exposing cytoplasm and nucleoplasm to the intercellular space (Figures 1E–1H). To our knowledge, this is the first published description of simultaneous rupture of the NE and the plasma membrane, and we suggest naming this cellular pathology PNH. For detailed characterization of this phenomenon, we performed extensive analyses of electron microscopy imagery of migrating neurons in CB_1_R^−/−^ embryos, wildtype control mouse embryos and embryos after *in utero* application of CB_1_R agonists CP-55940 and WIN 55,212-2 at different doses. Using three-dimensional (3D) reconstruction, we identified 122 nuclear hernias (including 65 NE ruptures and 57 PNHs). Morphometric analyses showed that volume of the streams of herniated chromatin varied from 0.02 to 1.63 μm^3^ (Table S1). Average volumes of herniated chromatin differed between the experimental groups showing a maximum (0.62 ± 0.04 μm^3^) for neurons with PNH from CB_1_R^−/−^ embryos (Figure 2A). In some analyzed cells, the PNH stream was narrow and reached the length of several microns (Figure S1). Although the molecular mechanism of PNH remains unclear, the large volume and length of the herniated chromatin streams indicate that at least one detail involved in the event is the presence of high intranuclear pressure during nuclear translocation in a tightly packed environment, such as a developing cerebral cortex.^72^

The vast majority of analyzed cells demonstrated an elongated shape, indicating active nuclear translocation in the moment of fixation. Another important piece of evidence for active migration is the intracellular position of the centrosome —the main center of cytoskeleton reformation— which periodically relocates in the direction of migration.^21^^,^^83^^,^^84^ By contrast, the anchoring of the mother centriole to plasma membrane and elongation of the cilia outside of the cell correlates with termination of migration.^85^ Accordingly, the majority of analyzed cells [total of 124 cells; of them, 37 cells from control group (Table S2), 23 cells from CB_1_R^−/−^ mice (Table S3), 32 cells from CP-55940 exposed embryos (Table S4); 32 cells from WIN 55,212-2 exposed embryos (Table S5)] contained free centrosomes in cytoplasm or showed initial steps of cilium development, such as mother centrioles connected to the cilial vesicles in cytoplasm or anchored to the plasma membrane on occasion producing short procilium. Moderately developed cilia (length of the axoneme ∼1 μm) were identified only in 4 cells from CP-55940 group (Table S4) and 6 cells from WIN 55,212-2 group (Table S5). Therefore, most if not all analyzed cells were in process of active migration in the moment of fixation, and their nuclei, likely exposed to high pressure, were predisposed to NE rupture.

### NE ruptures and PNHs are frequent in CB_1_R^−/−^ embryos and wildtype embryos exposed to CB_1_R agonists

**Figure 3:**
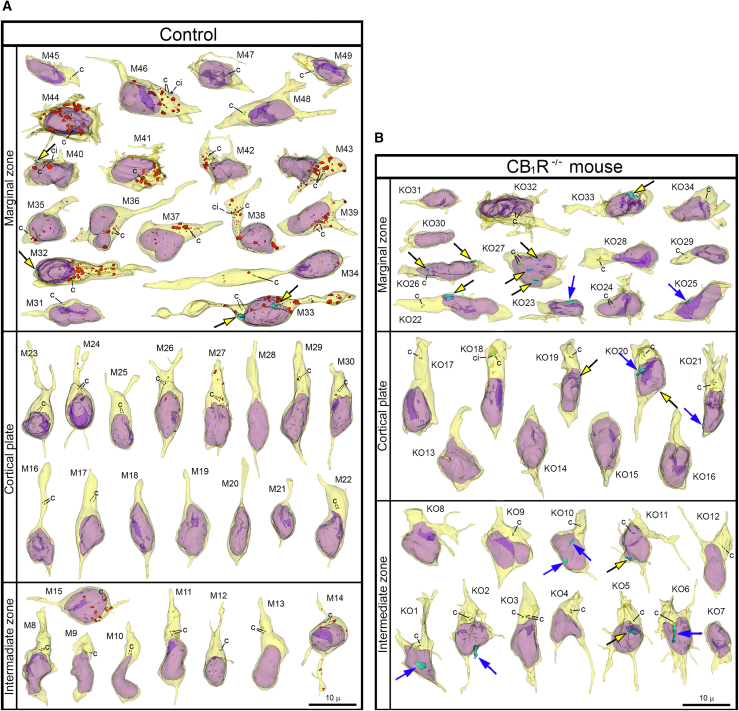
Collage of 3D reconstructions of migrating neurons from wildtype control embryos and CB1R−/− mouse embryos (A and B) Among 42 analyzed cells from control embryos, nuclear hernias are detected in 3 cells (A). Among 34 cells from CB1R−/− embryos, nuclear hernias are detected in 15 cells (B). Herniated chromatin streams are shown in light blue, with examples resulting from NE ruptures pointed to using black arrows with yellow heads, and streams producing PNHs are pointed to with blue arrows. The cells are positioned with pia-directed segments facing up and the ventricular surface-directed segments facing down. See Tables S2 and S3 for morphometric characteristics of each cell. Reconstructed cell bodies with proximal segments of processes are shown in semi-transparent yellow, and nuclei are shown in semi-transparent violet. Most of the identified processes are incompletely reconstructed because of truncation in the serial sections. Anti-CB1R immunoreaction depositions in wildtype embryos are depicted in red. Most of the interneurons tangentially migrating through the marginal zone show high CB1R content, although cells #M45 and M47-M49 are immunonegative in the reconstructed segments. Centrioles (c) are depicted in green, procilia (ci) – in blue. Scale bars are valid for all the cells. Images of the cells M8-M34 and KO1-KO16 were published in our previous article 21 (CC BY http://creativecommons.org/licenses/by/4.0).

**Figure 4:**
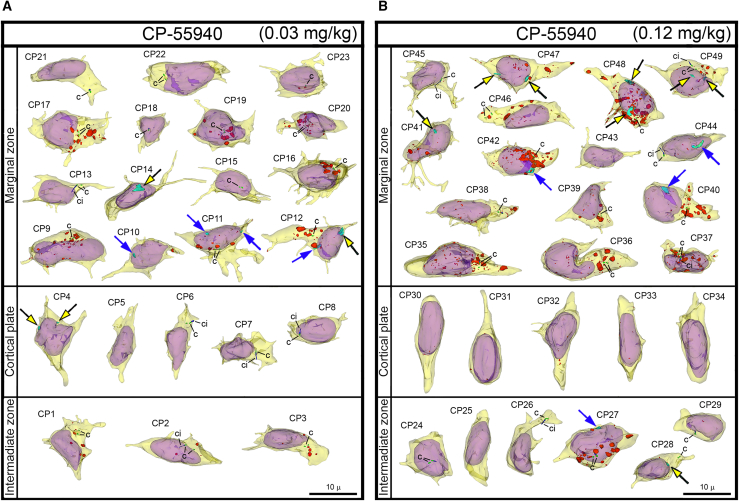
Collage of 3D reconstructions of migrating neurons from CP-55940 exposed embryos (A and B) Among a total of 49 analyzed cells, nuclear hernias were detected in 5 cells in the embryos exposed to low dose (A) and 9 cells in the embryos exposed to high dose of CB1R agonist CP-55940 (B). Herniated chromatin streams are shown in light blue, with streams resulting from NE ruptures pointed to using black arrows with yellow heads, and streams producing PNHs pointed to with blue arrows. The cells are positioned with pia-directed segments facing up and the ventricular surface-directed segments facing down. See Table S4 for morphometric characteristics of each cell. Reconstructed cell bodies with proximal segments of processes are shown in semi-transparent yellow, and nuclei are in semi-transparent violet. Most of the identified processes are incompletely reconstructed because of truncation in the serial sections. Anti-CB1R immunoreaction depositions are depicted in red. Centrioles (c) are depicted in green, procilia (ci) – in blue. Scale bars are valid for all the cells.

**Figure 5:**
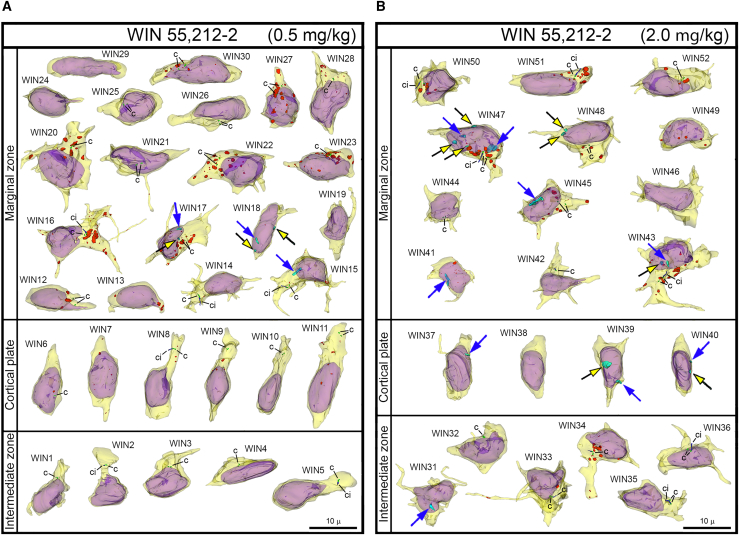
Collage of 3D reconstructions of migrating neurons from WIN 55,212-2 exposed embryos (A and B) Among a total of 52 analyzed cells, nuclear hernias are detected in 3 cells in the embryos exposed to low dose (A) and 9 cells in the embryos exposed to high dose of CB1R agonist WIN 55,212-2 (B). Herniated chromatin streams are shown in light blue, with the streams resulting from NE ruptures pointed to with black arrows with yellow heads, and streams producing PNHs pointed to with blue arrows. The cells are positioned with pia-directed segments facing up and the ventricular surface-directed segments facing down. See Table S5 for morphometric characteristics of each cell. Reconstructed cell bodies with proximal segments of processes are shown in semi-transparent yellow, and nuclei are shown in semi-transparent violet. Most of the identified processes are incompletely reconstructed because of truncation in the serial sections. Anti-CB1R immunoreaction depositions are depicted in red. Centrioles (c) are depicted in green, procilia (ci) – in blue. Scale bars are valid for all the cells.

For objective estimation of the frequency of chromatin herniation, we performed complete 3D reconstruction of randomly selected nuclei and cell bodies of migrating neurons from CB_1_R^−/−^ mouse embryos, wildtype control embryos and embryos exposed to CB_1_R agonists CP-55940 and WIN 55,212-2 at different doses (Figure 2B). Out of a total of 42 reconstructed cells from control embryos, only 3 cells showed NE ruptures, and none had PNH (Figure 3A; Table S2). In contrast, among 34 analyzed cells from CB_1_R^−/−^ embryos, 15 cells were herniated (of which 7 showed one or several NE ruptures and 8 cells showed at least one PNH; Figure 3B; Table S3). Application of two different CB_1_R agonists produced similar chromatin herniation, the extent of which was dose dependent. After injection of CP-55940 at low dose (0.03 mg/kg body weight), we detected 5 herniated cells (2 cells with NE ruptures and 3 cells with PNHs) out of a total of 23 analyzed cells, whereas the high dose (0.12 mg/kg) produced 9 herniated cells (5 cells with NE ruptures and 4 cells with PNHs) out of a total of 26 analyzed cells (Figure 4; Table S4). In the embryos exposed to low dose (0.5 mg/kg) of WIN 55,212-2, only 3 cells out of 30 analyzed cells showed hernias (all 3 cells showed at least one PNH), whereas the high dose (2.0 mg/kg) produced 1 cell with NE ruptures and 8 cells with PNHs out of a total of 22 analyzed cells (Figure 5; Table S5). Elevated frequencies (more than 40%) of herniated cells (NE ruptures and PNHs combined) were observed in the marginal zone of CB_1_R^−/−^ embryos and wildtype embryos exposed to high doses of both CB_1_R agonists. High frequencies of herniated cells were also detected in the intermediate zone and the cortical plate of CB_1_R^−/−^ embryos and wildtype embryos exposed to the high dose of WIN 55,212-2 (Figure 2B). Thus, we observed increased occurrence of NE ruptures and PNHs in all analyzed populations of migrating neurons – in both, the projection neurons migrating vertically through the developing cerebrum and in the horizontally migrating interneurons (Figure 2B). It is notable that profound effects to the frequency of herniated cells were produced by the opposite actions – knock-out of CB_1_R and its stimulation by agonists. This indicates that deviations from optimal functionality of the endocannabinoid system into either direction may affect the welfare of the nucleus and provoke chromatin herniation.

### Relationship of chromatin herniation and expression of CB_1_R in wildtype embryos

In control, CP-55940 and WIN 55,212-2 groups combined, a total of 29 herniated cells (NE ruptures and PNHs combined) were identified. Of those, 22 were located in the marginal zone (36% out of the 61 analyzed marginal zone cells), and only 7 were located in the intermediate zone or cortical plate (11% out of the 63 analyzed intermediate zone and cortical plate cells). This corresponds with a concentration of developing CB_1_R-expressing interneurons in the marginal zone and lower accumulation of CB_1_R in the cell bodies of migrating projection neurons.^21^^,^^78^ To further investigate if emergence of the chromatin hernia may be related to the level of CB_1_R accumulation, we estimated the amount of anti-CB_1_R immunolabeling end-product depositions in the herniated and non-herniated cells from all groups of wildtype embryos. Among 22 herniated cells from the marginal zone, 16 demonstrated visually high or moderate CB_1_R levels while 6 cells showed little to no CB_1_R immunolabeling in the reconstructed cell segments. Accordingly, out of 7 herniated cells from the intermediate zone and cortical plate, only 1 contained high levels of CB_1_R (cell CP27, which showed horizontal phenotype and may be an interneuron tangentially migrating through the intermediate zone), while 6 cells showed little to no CB_1_R immunolabeling (Table S6). While the applied method of estimation of CB_1_R content had several limitations (e.g., CB_1_R immunolabeling may be underestimated because of incomplete reconstruction of the cells), our observations indicate some degree of relationship between chromatin herniation and CB_1_R accumulation in the cell bodies. At the same time, this relationship may have probabilistic character as many CB_1_R-expressing neurons do not display hernias (Figures 4 and 5). Thus, our study revealed more frequent chromatin herniation in the CB_1_R-expressing interneurons rather than in the projection neurons in wildtype mouse embryos. Our immunohistochemical analyses show the probability (rather than unequivocal proof) of a link between chromatin herniation and the amount of CB_1_R in the cell bodies.

### Chromatin herniation correlates with mitochondrial fission in CB_1_R^−/−^ embryos

**Figure 6:**
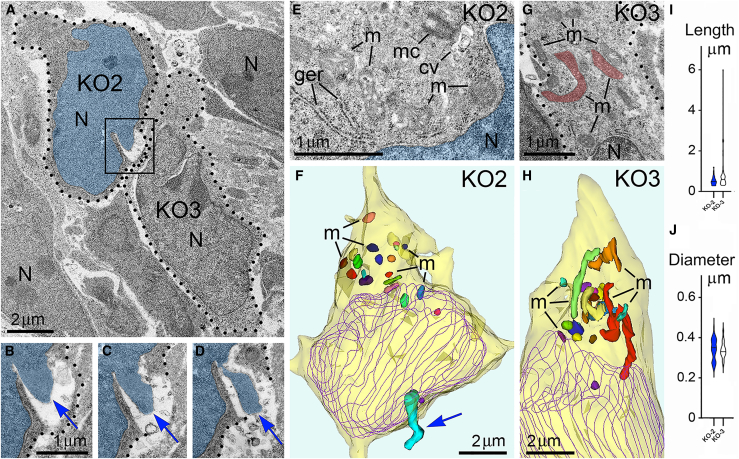
Adjacent herniated and non-herniated neurons show distinct ultrastructural characteristics of mitochondria (A) Low power micrograph of two neurons from the intermediate zone of CB1R−/− embryo, also shown in Figure 3 as cells number KO2 and KO3 (dotted lines designate their plasma membranes). Cell KO2 exhibits PNH whereas KO3 shows an intact nucleus. (B–D) Serial high power images of PNH from the framed area in (A). Notice the continuum of the chromatin stream (blue arrows) herniated from the nucleus (pseudo-colored blue) and penetrating the intercellular space as evidenced by the interruption of the plasma membrane (dotted lines) in (C) and (D). Scale bar in (B) is valid for (C) and (D). (E–H) High power images (E) and (G) and 3D reconstructions (F) and (H) of the cells KO2 and KO3. 20 randomly selected mitochondria from each cell are shown in different colors in the 3D reconstructions. Cell KO2 exposing PNH (blue arrow) contains mostly short or spherical mitochondria, whereas several mitochondria from cell KO3 are long, for example, the branching mitochondrion pseudo-colored in red in the electron micrograph (G) and 3D reconstruction (H). Nuclei profiles from every 4th serial section are traced in violet. Abbreviations: cv, cilial vesicle; ger, granular endoplasmic reticulum; m, mitochondria, mc, mother centriole; N, nucleus. (I and J) Estimation plots of lengths and diameters for mitochondria from cells KO2 and KO3. Violins of cell KO2 are colored blue. Horizontal lines in every violin indicate average length and diameter. For comparison of the estimation plots for mitochondria of all analyzed cells from CB1R−/− and control embryos see Figure S2.

**Figure 7:**
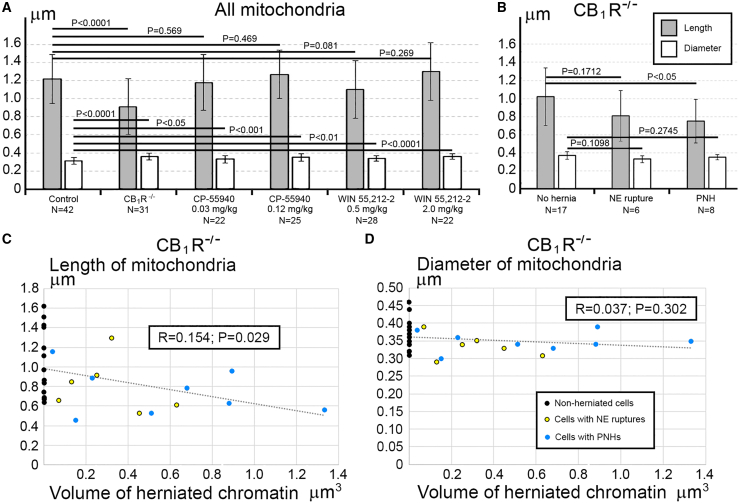
Morphometric analysis identifies reduction of mitochondria length in CB1R−/− embryos (A) T-test shows statistically significant reduction of the length of mitochondria from CB1R−/− embryos in comparison with the control group, whereas other groups show an absence of significant difference. Minor statistically significant (T-test) increase of mitochondrial diameters in CB1R−/−, CP-55940 and WIN 55212-2 groups in comparison with the control group may reflect swelling of a fraction of mitochondria. (B) In the CB1R−/− group, mitochondrial length shows statistically significant (T-test) reduction between ‘No hernia’ and PNH subgroups. The data in (A) and (B) are represented as the mean of average mitochondrial length and diameter from each cell at certain experimental conditions ±SD. N represents number of analyzed cells. (C and D) In the CB1R−/− group, the mitochondrial length (but not the diameter) shows statistically significant negative correlation (Pearson’s test) with the volume of herniated chromatin. Average length and diameter of the mitochondria from each cell is shown on the *y* axis in (C) and (D), respectively. Volumes of herniated chromatin streams are shown on the *x* axis. Morphometric characteristics of mitochondria from each analyzed cell are shown in Tables S2, S3, S4, and S5 and Figures S2, S3, and S4.

Morphology of mitochondria may be useful for quantitative characterization of cellular functionality. For example, prevalence of mitochondrial fission – as evidenced by their shorter length – reflects cellular disorder.^86^^,^^87^^,^^88^^,^^89^ Here, we observed that cells from the same sample may demonstrate dramatic ultrastructural differences – short mitochondria were found in the cells with PNH while adjacent non-herniated cells show normally sized mitochondria (Figure 6). These observations inspired our extensive morphometric analysis of the 3D reconstructed mitochondria, which identified morphologic heterogeneity of mitochondrial length and diameter in migrating neurons from all studied experimental groups (Tables S2, S3, S4, and S5; Figures S2, S3, and S4). Namely, in Control, CP-55940 and WIN 55212-2 groups, we detected similar fluctuations in mitochondrial length from ∼0.3 to ∼8 μm, and fluctuations of diameter from ∼0.2 to ∼0.6 μm, whereas in CB_1_R^−/−^ group, the length fluctuated between ∼0.3 and ∼6 μm, and diameter fluctuated from ∼0.1 to ∼0.8 μm. Analysis of average mitochondrial length showed a statistically significant decrease of mitochondrial length only in the CB_1_R^−/−^ group in comparison with the control group—this suggested up-regulation of mitochondrial fission in CB_1_R^−/−^ embryos (Figure 7A). An unpaired T-test of variances of average mitochondrial diameters showed a minor statistically significant increase in CB_1_R^−/−^, CP-55940 and WIN 55212-2 groups in comparison with the control group—this may indicate a slight swelling of a fraction of mitochondria after disruption of the endocannabinoid system (Figure 7A). However, while we saw minimal differences between the averages, certain cells showed a greater range in diameter measurements (Figures S2, S3, and S4). Accordingly, F-tests to compare variances in diameter and length showed no significant differences between these groups. We also analyzed mitochondrial morphometric characteristics in the experimental groups, separating them into three subgroups based on absence of the hernia (No hernia), or presence of a certain type of hernia (NE rupture or PNH). Average mitochondrial length showed statistically significant reduction in T-test between “No hernia” and PNH subgroups in CB_1_R^−/−^ embryos — this indicated an association of PNH with mitochondrial fission (Figure 7B). Moreover, the mitochondrial length (but not diameter) negatively correlated with the volume of herniated chromatin in the cells with NE ruptures and PNHs in CB_1_R^−/−^ embryos (Figures 7C and 7D). In CP-55940 and WIN 55,212-2 exposed embryos, variations of mitochondrial morphology between “No hernia”, NE rupture and PNH subgroups, as well as correlations of mitochondrial length and diameter with the volume of herniated chromatin, were not statistically significant (data not shown). Thus, at least in the CB_1_R^−/−^ embryos, emergence of PNHs correlates with mitochondrial fission — an ultrastructural feature associated with moderate cell disorder.^86^^,^^87^^,^^88^^,^^89^ However, the massive mitochondrial swelling that was documented in previous studies to be irreversible and foreshadowing cell death through a necrotic mechanism^86^^,^^88^ was not identified in this study.

### Probable recovery of the cells with PNH

**Figure 8:**
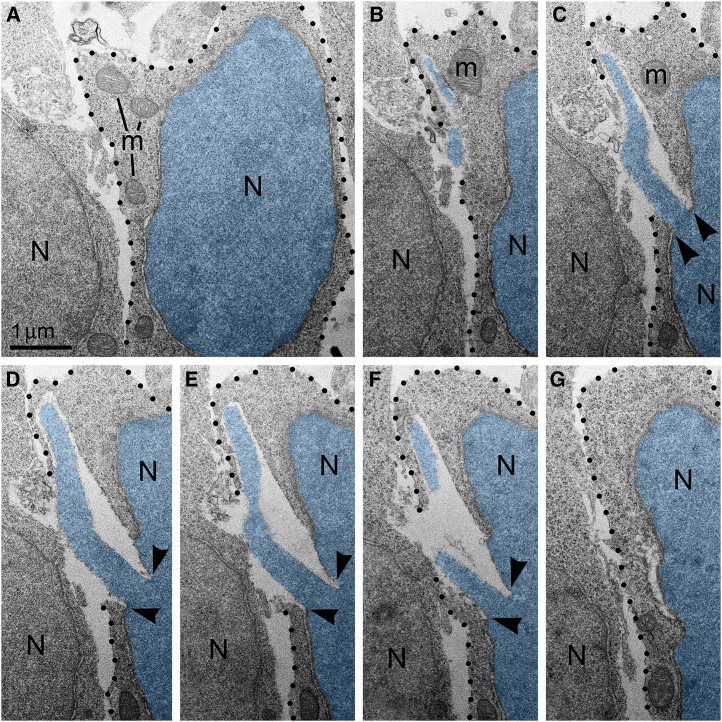
An example of plasma membrane rupture blocked by adjacent cell (A–G) Serial electron micrographs depict a neuron with PNH from the cortical plate of wildtype mouse embryo exposed to high dose (2.0 mg/kg) of CB1R agonist WIN 55,212-2. The nucleus and PNH stream are pseudo-colored blue. Points of interruption of NE are indicated with arrowheads. The plasma membrane is designated with dotted lines. Although the nuclear hernia produces a large rupture of the plasma membrane, the cellular cytoplasm does not show evidence of degradation and mitochondria do not show evidence of swelling. Scale bar in (A) is valid for all. Abbreviations: N, nucleus; m mitochondria.

Isolation of the cellular inner content by an intact plasma membrane is among the fundamental criteria of a living cell. High frequency of cells with ruptured plasma membrane in certain experimental conditions (Figure 2) suggests the death of many cells. Surprisingly, we did not observe cellular remnants or cells with ultrastructural features of necrotic, apoptotic or autophagy type degradation in the analyzed embryos. Instead, a fraction of the cells showed reduction of mitochondrial length — evidence of mitochondrial fission and cell disorder (Figures S2, S3, and S4). Different time courses of NE rupture (likely occurring in the span of seconds^57^^,^^60^^,^^61^^,^^62^^,^^63^) and mitochondrial reaction (delay in the span of hours^88^) may partially explain the divergence between emergence of PNH and reaction of organelles in the embryos temporarily exposed to CB_1_R agonists. Nevertheless, the absence of a considerable number of dead cells in CB_1_R^−/−^ embryos, which constantly experience disorder of endocannabinoid signaling, raises a supposition that PNH may be repaired rather than always be fatal for the cell. This observation aligns with the fact that CB_1_R^−/−^ mice generally do not exhibit obvious behavioral phenotypes.^90^ In accordance, partial recovery of cells after NE rupture is known.^64^^,^^76^ Surviving neurons were reported after transection of the axons, but not after damage of the cell bodies.^91^ Here, we considered morphological features that can provide possible recovery of migrating neurons with PNH. Namely, for a fraction of the cells with PNH, the area of the plasma membrane rupture was closely surrounded by adjacent cell bodies or processes (Figure 8), which may contribute to decreasing the flow of cytoplasm and effectively blocking the leak. We observed blocking of the plasma membrane rupture by adjacent cells at 25 PNHs among a total of 57 examples analyzed. Of them, 4 blocked PNHs out of 13 analyzed (30.8%) were identified in CB_1_R^−/−^ embryos, 13 blocked out of 20 analyzed (65.0%) — in CP-55940 exposed embryos and 8 blocked out of 24 analyzed (33.3%) — in WIN 55,212-2 exposed embryos (Table S1). Blocking of the plasma membrane rupture by adjacent cells, together with a possible delay of mitochondrial reaction, may explain our observations of normally sized mitochondria in several 3D reconstructed cells with PNH. At the same time, plasma membrane blocking may be ineffective for other cells that showed considerable reduction of mitochondrial length (Figures S2, S3, and S4). Thus, although death of cells with ruptured NE and plasma membrane is probable, PNH in mouse embryo brains may be non-fatal for a fraction of the affected cells. This suggests the possibility of recovery for catastrophically damaged neuronal cell bodies, which has not been demonstrated previously.

## Discussion

Here, we report generation of powerful chromatin streams breaking NE together with the plasma membrane, which we suggest naming PNH. About 40% of randomly analyzed cells among migrating projection neurons and interneurons demonstrated NE ruptures or PNHs in CB_1_R^−/−^ embryos and in wildtype embryos shortly exposed to high doses of synthetic agonists of CB_1_R – 0.12 mg/kg of CP-55940 or 2.0 mg/kg of WIN 55,212-2. This indicates that deviation from optimal functioning of the endocannabinoid system in either direction —knock-out of CB_1_R or its temporary overstimulation— increases likelihood of altered cellular nucleus and provoke chromatin herniation in a similar way. Among the embryos exposed to aforementioned experimental conditions, CB_1_R^−/−^ embryos showed maximal ultrastructural pathology in terms of high frequency of herniated cells, high volume of chromatin streams, reduced average length of mitochondria in all the cells, more pronounced reduction of average length of mitochondria in the cells with PNHs, and negative correlation of the mitochondrial length with the volume of herniated chromatin in the cells with NE ruptures and PNHs. Our finding of experimental conditions increasing the frequency and power of chromatin herniation in migrating cells may be instrumental for further investigation of mechanisms of NE rupture and repair as well as for study of the role of the endocannabinoid system in cytoskeleton functionality.

As described here, PNHs catastrophically break isolation of the inner content of the cell from the environment. Cells exposed to extreme physical, chemical, or mechanical stimuli may immediately lose their structural integrity and die in an uncontrollable manner termed ‘accidental cell death’, which is opposite to mechanisms of programmed cell death such as apoptosis, autophagy, or necrosis-type cell death.^91^^,^^92^^,^^93^^,^^94^ Cases of accidental cell death are virtually undetectable using biochemical or immunochemical methods, for example, TUNEL staining, caspase immunolabeling, or labeling of autophagy markers. Electron microscopy visualizes organelles regardless of their functional conditions and can detect dead cells or cellular remnants. Surprisingly, we did not observe considerable numbers of dead cells in the samples containing numerous cells with herniated nuclei. This suggests an opportunity for repair of NE and plasma membranes in at least a fraction of the affected cells, providing their survival of the initial breach. Indeed, NE ruptures may be repaired without loss of cell viability.^57^^,^^60^^,^^61^^,^^63^ Repair of large ruptures of the plasma membrane was shown in muscle cells, oocytes, epithelial cells, invasive cancer cells and other cell types. Neurons may effectively restore transected axons, but recovery of mechanically damaged cell bodies was not documented.^95^^,^^96^ The initial repair steps include plugging the membrane rupture with conglomerates of vesicles or membrane whorls that restrict the leak of cytoplasm providing time for reconstruction of intact plasma membrane.^95^^,^^96^^,^^97^^,^^98^ The major trigger for the signaling cascade that precedes membrane sealing is the influx of Ca^2+^ through the membrane disruption. Subsequent vesicle deposition and fusion involve complex Ca^2+^-dependent pathways acting through the cAMP signaling cascade, cytoskeleton reconstruction, SNAP receptors, the phospholipase enzymes, calpain proteases and others. The membrane patch normally serves as a temporary barrier and is subsequently remodeled or removed via exocytic or endocytic machinery.^95^^,^^97^^,^^98^^,^^99^ We observed analogous cases when adjacent cells surround the plasma membrane rupture, apparently delaying degeneration of the damaged cell, as identified by a reduction in morphological disorder of mitochondria. Lack of dead cells in samples containing numerous neurons with catastrophically ruptured plasma membranes is the first evidence for survival of severely damaged cell bodies of mammalian neurons. Deeper study of this phenomenon may become instrumental for increasing regenerative capacity of neuronal cell bodies after traumatic brain injury, ischemic conditions and during neurodegenerative diseases.^91^^,^^96^^,^^97^^,^^98^^,^^99^^,^^100^

The early expression of CB_1_R indicates its probable involvement in prenatal and postnatal brain development.^20^^,^^78^ Numerous studies indicate that prenatal inhibition or overstimulation of the endocannabinoid system may provoke long-term consequences in the offspring, for example, altered breathing, disturbed suckling, and memory deficit linked to disorder of glutamate-ergic neurons,^15^^,^^45^^,^^101^^,^^102^^,^^103^^,^^104^ but the underlying mechanisms remain enigmatic in many aspects. Our data indicate that disorder of the endocannabinoid system may provoke NE ruptures and PNHs in migrating neurons, a fraction of which also acquire moderate ultrastructural pathology. Although we did not observe completely destroyed cells, the present study does not assume a complete recovery of all cells experiencing NE ruptures or PNHs. More likely, temporary damage of NE and plasma membrane provokes a delay or disorientation of cell migration. This may be the reason for previously identified brain dysfunction in CB_1_R^−/−^ mice or cognitive deviations in the animals and humans exposed to cannabis during development that, nevertheless, do not exhibit obvious behavioral phenotypes or evidence of teratogenic effects of the cannabinoids.^14^^,^^15^^,^^45^^,^^90^^,^^101^^,^^102^^,^^103^^,^^104^ Our finding opens an opportunity for further investigation if temporary disorder of the endocannabinoid system, for example, in cases of recreational cannabis use by pregnant women, may provoke accidental death or malfunction of migrating neurons.

Judging from the large volume and length of the herniated chromatin streams, the NE ruptures and PNHs observed here may be consequences of increased intranuclear pressure, which occurs during nuclear translocation through tightly packed tissue such as the developing mammalian cerebrum. The generally accepted model of NE rupture and chromatin herniation presumes participation of cytoskeleton and nucleoskeleton in the increasing of intranuclear pressure.^59^^,^^72^ Chronic treatment of adult rats with WIN 55,212-2 increased expression of neurofilaments Nf-160 and Nf-200, and microtubule-associated protein-2 (MAP-2). Meanwhile, CB_1_R^−/−^ mice demonstrated lower expression of the neurofilaments and MAP-2, along with reduced dendritic arborization in the hippocampus.^105^^,^^106^^,^^107^ Such deviated expressions of the cytoskeletal components after stimulation or knock-out of CB_1_R constitute evidence of its participation in neuronal cytoskeleton consolidation. Link of endocannabinoid system and particularly CB_1_R with the function of actin filaments was also demonstrated in the context of axonal pathfinding. This mechanism involves CB_1_R internalization from filopodia and chemorepulsion of the axonal growth cones by activating RhoA GTPases (small guanosine triphosphatases).^24^^,^^25^ RhoA GTPases are known for their role as molecular switches transducing extracellular stimuli to the actin cytoskeleton.^108^^,^^109^ Thus, the unknown role of CB_1_R in the developing brain may include maintaining optimal function of the cytoskeleton, which is crucial for translocation of neuronal cell bodies.

Our findings may also have applications for cancer research. Although NE rupture resulting in genomic instability might promote cancer progression, unstable NE also represents a particular weakness of metastatic cancer cells. Accordingly, NE ruptures detected in many cancer cell lines during cell migration through tightly constricted areas often result in cell death.^57^^,^^58^^,^^60^^,^^66^ Conditions that have potential to increase the probability of NE rupture and death of metastatic cells are considered potent for cancer treatment.^47^^,^^57^ We suggest investigating the conditions of PNH generation for targeting metastatic cancer cells. Such conditions may include manipulations of the endocannabinoid system and probably other molecular mechanisms, to increase the frequency and power of NE ruptures. Numerous studies have shown capability of cannabinoids as anticancer agents in several disease models *in vitro* and *in vivo*. The observed effects of the cannabinoids’ application include inhibiting tumor growth and metastasis, reducing cell viability through promoting apoptosis, and inhibiting angiogenesis; but the molecular mechanisms at play were not identified in detail. Accordingly, no strong clinical trial data exists to confirm the pre-clinical evidence for cannabinoids’ anticancer effects.^38^^,^^39^^,^^40^^,^^41^^,^^43^^,^^110^^,^^111^^,^^112^ Our findings suggest a mechanism of cannabinoid action on the nuclei of migrating cells and may be instrumental for inducing breaks of the plasma membrane in metastatic tumor cells. This opens an opportunity for a new research direction that may result in development of therapeutic applications of cannabinoids for targeting cancer cells.

### Limitations of the study

In this article, we applied a method of 3D reconstruction of cells from a long series of ultrathin sections, which provides an opportunity for high resolution *in vivo* morphologic analysis of cells at certain developmental stages. Nevertheless, the application of electron microscopy as the only research method is among the limitations of this article. Confirmations using different methods of analysis are required. For example, it makes sense to develop equipment and procedures for *in vitro* study of cell migration through restricted space, suitable for detecting herniated nuclear material with light microscopy or other express methods. Another source of uncertainty in our findings is the fact that the analysis of CB_1_R was performed with only one immunolabeling method. Different CB_1_R isoforms, heterodimers, or other receptors, such as cannabinoid type 2 receptor, G protein-coupled receptor 55 and transient receptor potential vanilloid-1 (if present in the developing cerebrum, which awaits conformation^14^) may respond to synthetic cannabinoids. Moreover, genetic knockout models such as CB_1_R^−/−^ mouse preserve the opportunity for developmental compensation that may partially challenge the conclusion. Analysis of conditional knock-out mice can mitigate this problem.

## Resource availability

### Lead contact

Requests for further information, resources, or reagents should be made to Dr. Yury M. Morozov (yury.morozov@yale.edu).

### Materials availability

No novel reagents were generated by this study. CB_1_R^−/−^ *(Cnr1*^−/−^) mice were kindly provided by Dr. Ken Mackie, Indiana University.

### Data and code availability

• This study did not generate/analyze any datasets/code.
• Microscopy data reported in this paper will be shared by the lead contact upon request.
• Any additional information required to reanalyze the data reported in this paper is available from the lead contact upon request.

## Acknowledgments

We are grateful to Ekaterina Morozova and Mikhail Morozov for editorial work on the manuscript. This research was supported by the Kavli Institute for Neuroscience at Yale, and USA NIH NIDA grant DA023999 to P.R.

## Author contributions

Y.M.M., conceptualization, data curation, formal analysis, investigation, methodology, validation, visualization, and writing; P.R., funding acquisition, supervision, and validation.

## Declaration of interests

The authors declare no competing interests.

## STAR★Methods

### Key resources table

**Table:** 

REAGENT or RESOURCE	SOURCE	IDENTIFIER
**Antibodies**		

Guinea pig CB_1_R	Frontier Science Co. Ltd, Japan	Cat# CB1-GP-Af530

**Chemicals**		

WIN 55,212-2	Biomol International LP, Plymouth, PA, USA	Cat# CR-105
CP-55940	Sigma-Aldrich	Cat# C1112
3,3′-Diaminobenzidine	Thomas Scientific	Cat# C963P95
Ultrostain-2	Leica Biosystems	Cat# 16707235

**Experimental models: Organisms/strains**		

Mus musculus:C57BL/6	The Charles River Laboratory	N/A
Mus musculus:CD1-Cnr1^−/−^	Laboratory of Dr. K. Mackie	Ledent et al.^113^ MGI: 1857736

**Oligonucleotides**		

5′-CATCATCACAGATTTCTATGTAC-3′	IDTDNA	Parmentier-Batteur et al.^114^
5′-GAGGTGCCAGGAGGGAACC-3′	IDTDNA	Parmentier-Batteur et al.^114^
5′-AAGGAAGGGTGAGAACAGAG-3′	IDTDNA	Parmentier-Batteur et al.^114^
5′-GATCCAGAACATCAGGTAGG-3′	IDTDNA	Parmentier-Batteur et al.^114^

**Software and algorithms**		

Reconstruct 1.1.0.0.	http://www.bu.edu/neural/Reconstruct.html	Fiala^115^; Fiala and Harrs^116^
GraphPad Prism 10.1.2	GraphPad Software, LLC	GraphPad.com RRID:SCR_002798

### Experimental model and subject details

#### Animals and genotyping

All animal protocols were compliant with the National Institutes of Health (USA) guidelines for animal care and use and were approved by the Institutional Animal Care and Use Committee of Yale University (Protocol #2018–10750.A3 approved 25 June 2020). Control wild-type mice and mice for prenatal stimulation of CB_1_R (see below) were obtained from a colony consisting of a stable strain of C57BL/6 mice. CB_1_R^+/+^ and CB_1_R^−/−^ mice were generated by breeding CB_1_R^+/−^ pairs in CD1 background as described previously.^113^ Breeding pairs were housed together on a 12-12-h light-dark cycle with food and water available *ad libitum*. Primers used for CB_1_R PCRs were 5′-CATCATCACAGATTTCTATGTAC-3′ and 5′-GAGGTGCCAGGAGGGAACC-3′, to amplify a 366 bp band from the wild-type allele and 5′-GATCCAGAACATCAGGTAGG-3′ and 5′-AAGGAAGGGTGAGAACAGAG-3′, for a 521 bp band from the mutant CB_1_R allele.

### Method details

#### Prenatal stimulation of CB_1_R

For prenatal stimulation of CB_1_R, we performed two intraperitoneal (IP) injections of wild type pregnant mice with synthetic agonists, WIN 55,212-2, or CP-55940 at low and high doses. First injection was done 24 h before sacrifice, and the second - 1 h before sacrifice the dams, followed by the embryo brains collection. The doses of WIN 55,212-2 - 0.5 mg per kg of body weight (mg/kg) and 2.0 mg/kg - were chosen on the basis of previous publications that showed the low dose to be non-toxic for the embryos and the high dose to have significant affection of reproduction parameters in cases of prolonged prenatal exposure.^101^^,^^102^ The doses of CP-55940 - 0.03 mg/kg and 0.12 mg/kg - were chosen on the basis of known high range of its effective doses in developing and adult brain.^12^^,^^117^^,^^118^^,^^119^ The drugs were solved in the vehicle containing 5% DMSO, 5% Tween 80 and saline and injected in the volume of 10 mL/kg body weight. Volume-equivalent vehicle was injected in the control pregnant mice. For the morphologic analysis, we used CP-55940 exposed embryos at the low dose at the embryonic day (E) 13 (*N* = 4); CP-55940 exposed embryos at the high dose at E12 (*N* = 4); WIN 55,212-2 exposed embryos at the low dose at E12 (*N* = 1), E13 (*N* = 3) and E16 (*N* = 1) and WIN 55,212-2 exposed embryos at the high dose at E13 (*N* = 4). The control group included embryos from the vehicle-injected pregnant mice at E12 (*N* = 1) and E16 (*N* = 3), as well as untreated embryos at E13 (*N* = 1) and E14 (*N* = 1). We did not observe systematic differences in the analyzed parameters between the vehicle-exposed and untreated embryos as well as between the embryos at different ages from same experimental group. Totally, 6 control embryos, 4 low dose CP-55940; 4 high dose CP-55940, 5 low dose WIN 55,212-2 and 4 high dose WIN 55,212-2 exposed embryos, as well as 4 CB_1_R^−/−^ embryos at E14 in CD1 background were used for below morphologic studies.

#### Electron microscopy and 3D reconstruction

Pregnant mice were anaesthetized with IP injection of sodium pentobarbital (3 mL/kg) or euthasol (0.5 mL/kg) and sacrificed with cervical dislocation. The embryos were removed from the uterus and their age was adjusted based on the anatomical landmarks. Embryos without apparent abnormalities were decapitated, brains were removed and immersed in the fixative containing 4% paraformaldehyde and 0.2% glutaraldehyde in 0.1M phosphate buffer (pH 7.4) for 3–4 days at 4°C. Coronal 100-mm-thick brain slices were cut with a vibratome and prepared for immunohistochemistry and electron microscopy as described below. After incubation with the CB_1_R antibodies (Frontier Science Co. Ltd; dilution 1:2000), the slices were extensively washed and immersed in a solution of biotinylated anti-guinea pig IgGs (Jackson Immunoresearch; Cat#706-065-148; 1:300) and the Elite ABC kit (Vector Laboratories, Burlingame, CA, USA; Cat# PK-6100; 1:300). Ni-intensified 3,3′-diaminobenzidine–4HCl (DAB-Ni) was used as a chromogen. Brain slices were post-fixed with 1% OsO_4_, dehydrated and embedded in Durcupan (EMS, Hatfield, PA, USA; Cat# 14610). Electron microscopy and 3D reconstruction from ultrathin sections were performed as described previously^21^^,^^88^ with the following minor modifications. Selected segments from the lateral neocortex were re-embedded into Durcupan blocks and cut by a Reichert ultramicrotome into 60-nm-thick sections. Series of 150–200 consecutive sections from occasionally taken segments of the intermediate zone, cortical plate, or marginal zone were collected on one-slot grids covered with Formvar or Butvar B-98 films (both from EMS, Hatfield, PA, USA), stained with Ultrostain-2 (Leica Biosystems, Danvers, MA, USA) and evaluated in JEM 1010 electron microscope (JEOL, Japan) equipped with Multiscan 792 digital camera (Gatan, Pleasanton, CA, USA) or Talos L120C electron microscope equipped with Ceta digital camera (ThermoFisher Scientific, Boston, MA). Serial images of randomly selected interphase cells were made with 12,000× or 15,000× magnification of JEM1010 or with 3,400× or 4,300× of Talos L120C electron microscope. 3D reconstructions of cell bodies, nuclei, centrioles, cilia and mitochondria and measurements of the volumes of the reconstructed objects were performed using Reconstruct 1.1.0.0. software (Boston, MA)^115^^,^^116^ publicly available at http://www.bu.edu/neural/Reconstruct.html.

#### Identification of chromatin herniations

Ruptures of the nuclear envelope (NE) and herniations of chromatin streams were identified in serial sections. The hernias that contained chromatin streams in cytoplasm while the plasma membrane stayed intact were identified as NE ruptures; the hernias pairing with local damage of the plasma membrane and extending the chromatin streams in the intercellular space were identified as piercing nuclear hernias (PNH). Cells showing both, NE rupture and PNH, were counted as belonging to the PNH group. PNHs with the plasma membrane rupture closely surrounded by adjacent cells in all serial sections were counted as blocked PNHs. Percentages of the cells with NE rupture and PNH were calculated among the cells randomly selected for serial photography and 3D reconstruction. The quantifications avoided subjectivity as the nuclear hernias were mostly local and identifiable in the analyzed cells during their 3D reconstruction, which was performed after serial photography of the cells was completed. All the serially photographed cells were included in the estimations of the frequencies of herniated cells. The hernias identified during the electron microscopy session were serially photographed for measurements of their volume and were excluded from the estimations of the frequencies of herniated cells. For volume measurement, herniated chromatin streams were traced in the serial sections along the periphery of the stream in cytoplasm and, in case of PNH, in the intercellular space, and along straight lines connecting points of interruption of the nuclear membranes. Values of volume measurements of the herniated chromatin streams for each experimental group were calculated as mean ± SEM.

#### Estimation of intracellular CB_1_R

Anti-CB_1_R DAB-Ni depositions in each cell were traced for 3D reconstruction and counted as “single” depositions if local staining were identified in at least 3 serial sections; merging conglomerates of the immunoreaction end-product around intracellular vesicles were counted as “globule” depositions.^21^ The degree of CB_1_R accumulation in the 3D reconstructed cell segments was estimated as low, middle or high.

#### Morphometry of mitochondria

Morphometric characterization of 3D reconstructed mitochondria was performed as we validated in our previous articles.^86^^,^^87^^,^^88^^,^^120^ Namely, the length of mitochondria was estimated in the 3D images. The root-mean-square diameter of the reconstructed mitochondria was calculated based on the volume formula for ellipsoids using their length as the longest diameter of the ellipsoid. To optimize the time-consuming 3D reconstruction and morphometrical analysis of mitochondria, we performed the following pilot test. We reconstructed and measured all available mitochondria (more than 100 in total) in two cells. Then in both cells, we randomly assigned all cellular mitochondria into subgroups containing 20 mitochondria and performed statistical comparison of the group with the subgroups. Unpaired T-test showed absence of statistically significant difference between the groups of all mitochondria and the subgroups containing 20 mitochondria from both analyzed cells. So, this pilot test showed that 20 randomly chosen mitochondria adequately characterize the mitochondrial system of a cell. In this article, we restricted the quantity of analyzed mitochondria to 40 per cell. For the cells containing less than 40 mitochondria in the serial sections, we reconstructed and analyzed all available mitochondria. Cells containing less than 10 completely reconstructed mitochondria were excluded from the quantitative analysis of mitochondria.

### Quantification and statistical analysis

Statistical analyses of mitochondrial morphometric parameters and generation of violin estimation plots were performed using GraphPad Prism 10.1.2 software. Values of mitochondrial length and diameter were expressed as mean ± SD for every analyzed cell. Values of mitochondrial length and diameter for each experimental group were calculated as mean ± SD from average length and diameter of each cell. Unpaired T-test and F-test statistical analyses of variances were applied for comparison of mitochondrial lengths and diameters between the experimental groups. The relationships between the quantified mitochondrial parameters and the volume of herniated chromatin were assessed by Pearson’s correlation. All *p* values were 2-tailed; the value of *p* < 0.05 was used as the threshold for significance.
