# Supplementary material for: Abnormalities of the endocannabinoid system produce piercing nuclear hernias in migrating cerebral neurons
Source: iScience. 2025 Jul 23;28(8):113188. doi: 10.1016/j.isci.2025.113188 (PMC12341634; doi:10.1016/j.isci.2025.113188)
Supplement: Document S1. Figures S1–S4 and Tables S1–S6 [file mmc1.pdf]

iScience, Volume *28*

Supplemental information

Abnormalities of the endocannabinoid system produce piercing nuclear hernias in migrating cerebral neurons

Yury M. Morozov and Pasko Rakic

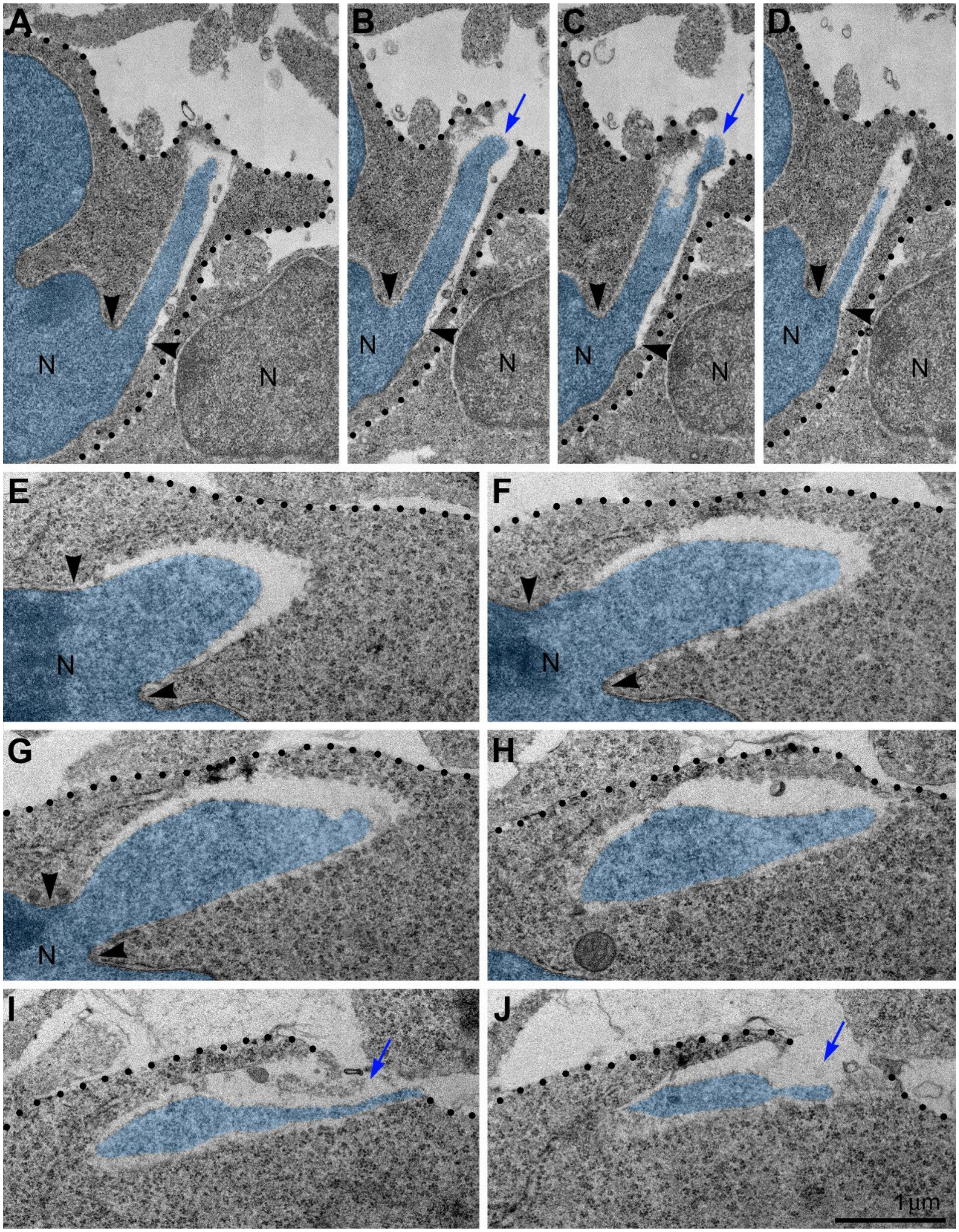

Figure S1. Long length and large volume of herniated chromatin streams are evidence of high intranuclear pressure destroying NE

(A-D) Serial micrographs of PNH in a neuron from the cortical plate of $CB_1R^{-/-}$ embryo show continuum of the chromatin stream expelled from the nucleus (pseudo-colored blue) and penetrating the intercellular space. Notice that the herniated chromatin stream extends about 2-µm away from the normal segment of the nucleus.

(E-J) Serial micrographs of PNH in a neuron from the cerebral intermediate zone of an embryo exposed to high dose (2.0 mg/kg) of WIN-55,212-2. Volume of the herniated chromatin stream was estimated in the complete series of sections as 1.63 μm^3. Blue arrows in (B), (C), (I) and (J) denote the interruption of the plasma membranes, which are designated with dotted lines. Points of NE interruption are indicated with arrowheads. Scale bar in (J) is valid for all. Abbreviation: N, nucleus.

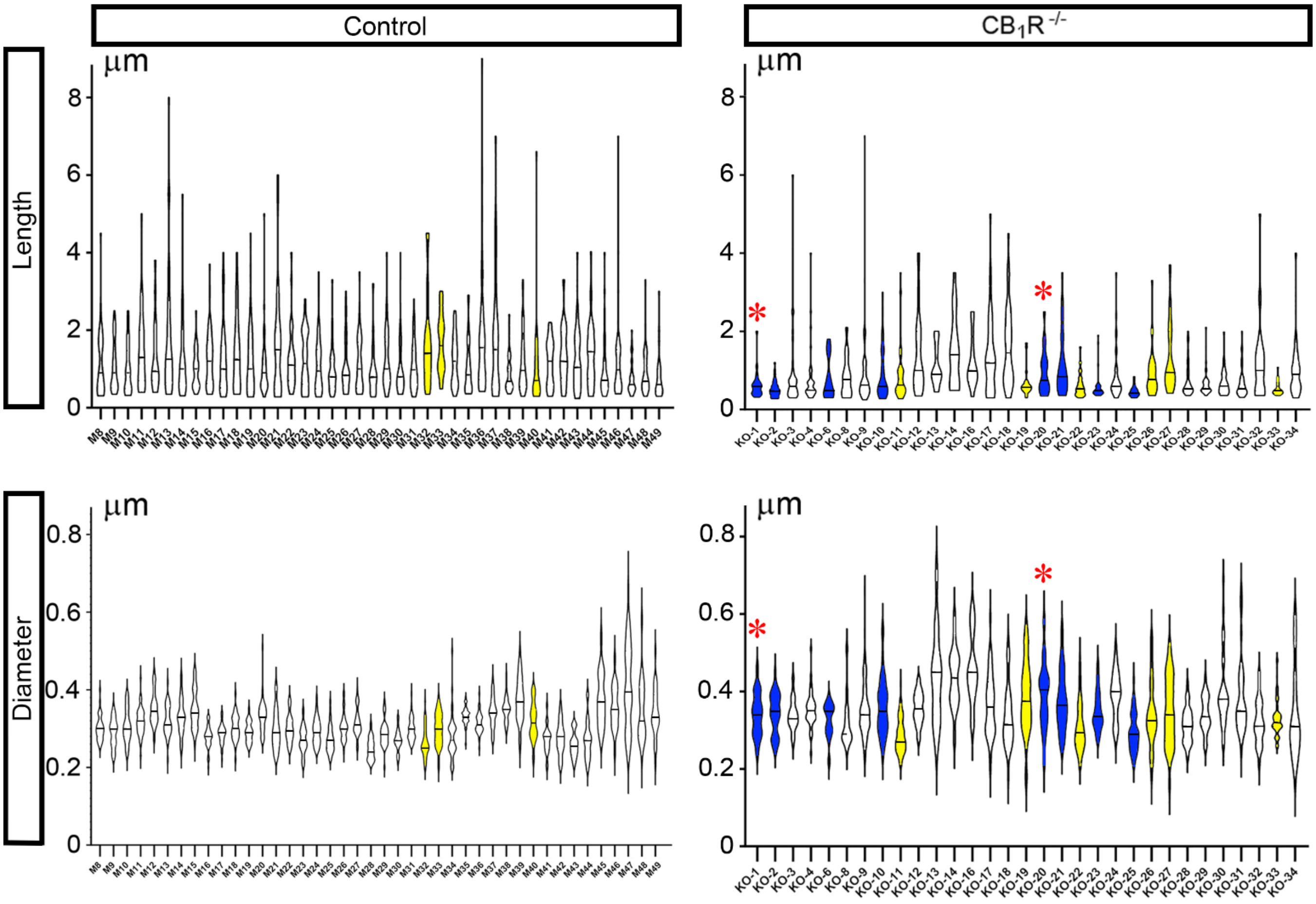

Figure S2. Estimation plots of length and diameter for mitochondria from all analyzed cells from wild type and $CB_1R^{-/-}$ embryos

Mitochondria from $CB_1R^{-/-}$ embryos are generally shorter and have more widely varied diameters, indicating increased mitochondrial fission and swelling. Violins of the cells with NE ruptures and PNHs are colored yellow and blue, respectively. PNH cells with the plasma membrane ruptures blocked by adjacent cells are indicated with red asterisks. Horizontal lines in every violin indicate average length and diameter. For comparison of the average morphometric characteristics see **Figure 7** in main text.

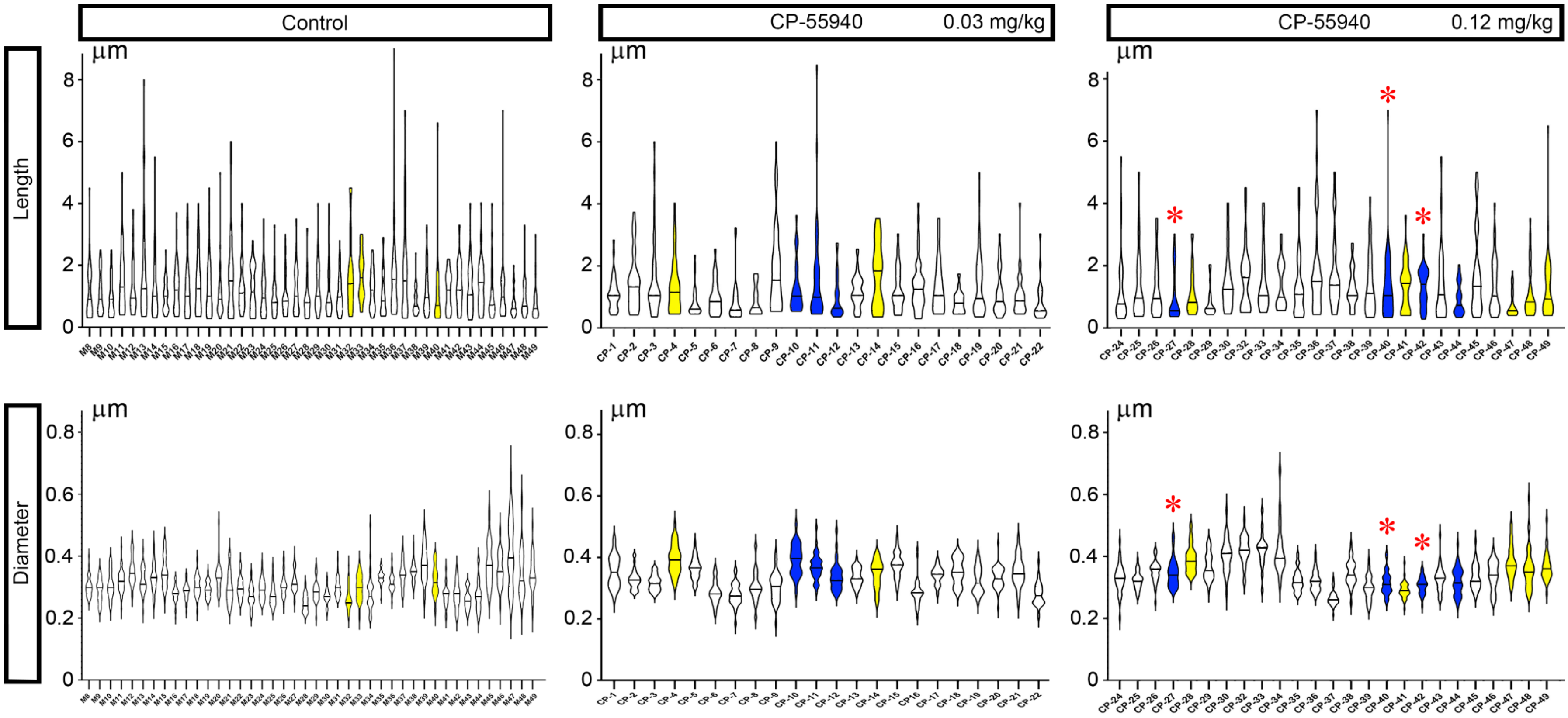

Figure S3. Estimation plots of length and diameter for mitochondria from all analyzed cells from control wild type embryos, and embryos exposed to CB_1R agonist CP-55940 at low and high doses

Variations of mitochondrial length and diameter are similar between control (repeated from **Figure S2**) and CP-55940 exposed embryos. Violins of the cells with NE ruptures and PNHs are colored yellow and blue, respectively. PNH cells with the plasma membrane rupture closely surrounded by adjacent cells are indicated with red asterisks. Horizontal lines in every violin indicate average length and diameter. For comparison of the average morphometric characteristics see **Figure 7** in main text.

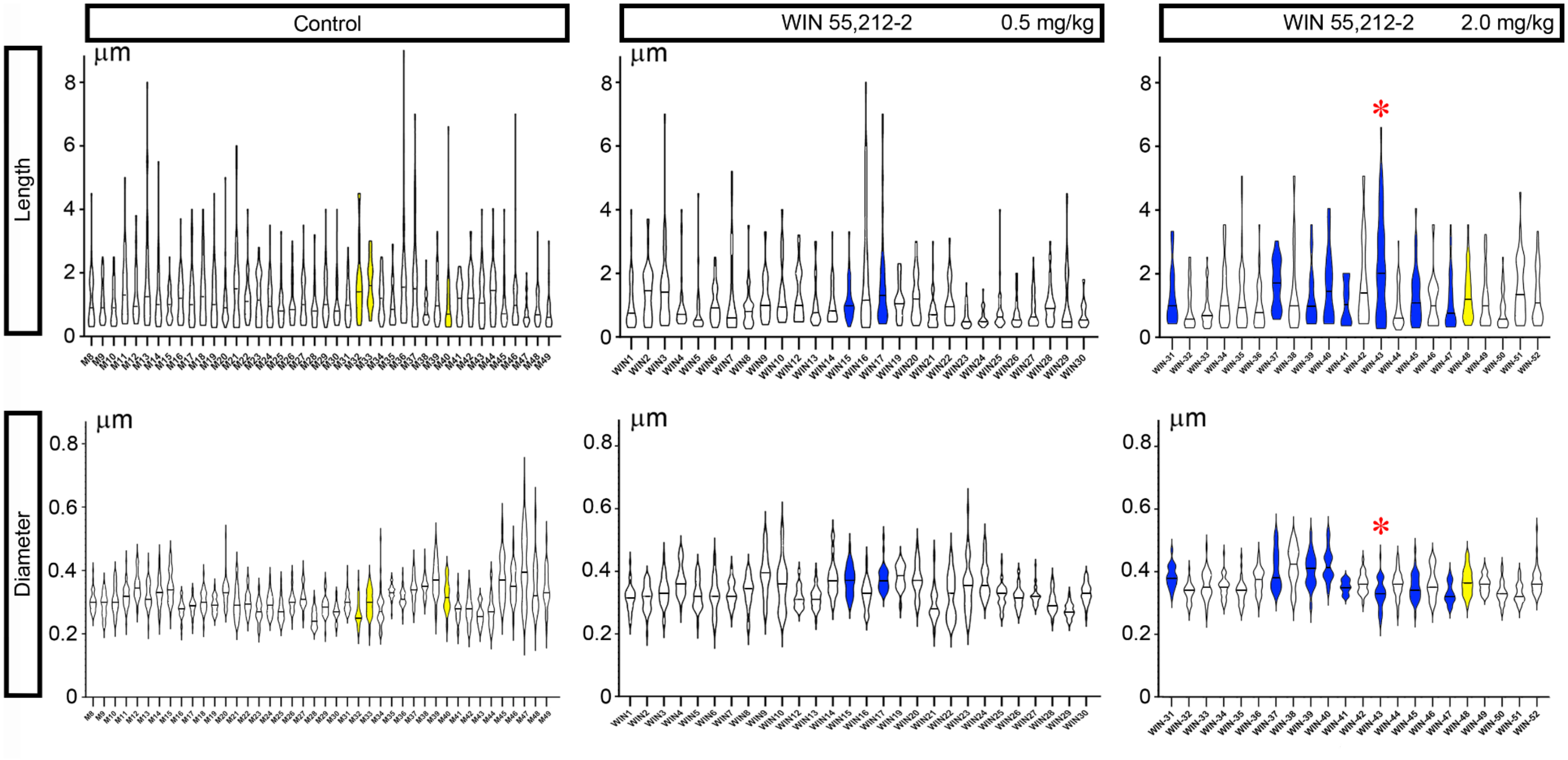

Figure S4. Estimation plots of length and diameter for mitochondria from all analyzed cells from control wild type embryos and embryos exposed to CB$_1$R agonist WIN 55,212-2 at low and high doses

Variations of mitochondrial length and diameter are similar between control (repeated from **Figure S2**) and WIN 55,212-2 exposed embryos. Violins of the cells with NE ruptures and PNHs are colored yellow and blue, respectively. PNH cell with the plasma membrane rupture closely surrounded by adjacent cells is indicated with a red asterisk. Horizontal lines in every violin indicate average length and diameter. For comparison of the average morphometric characteristics see **Figure 7** in main text.

Table S1.

Volume of herniated chromatin streams at different experimental conditions

Cell code (embryo age/#)	Cell body location	NE ruptures, μm^3	PNHs, μm^3
**WT Control**			
M32 (E16/#3)	MZ	0.07	
M33 (E16/#3)	MZ	0.45+0.63=1.08*	
M40 (E13/#1)	MZ	0.05	
N (of them blocked by adjacent cells)		4 (N/A)	0 (0)
MEAN ± SEM		0.30 ±0.07	-
**$CB_1R^{-/-}$**			
KO-1 (E14/#1)	IZ		0.88**
KO-2 (E14/#1)	IZ		0.51
KO-5 (E14/#1)	IZ	0.34	
KO-6 (E14/#1)	IZ		0.68
KO-10 (E14/#2)	IZ		0.10+0.13=0.23
KO-11 (E14/#2)	IZ	0.16	
Cell not numbered (E14/#1) †	IZ		0.34
Cell not numbered (E14/#1)	IZ		1.50**
Cell not numbered (E14/#1)	IZ		0.60
Cell not numbered (E14/#2)	IZ	0.11	
KO-19 (E14/#1)	CP	0.07	
KO-20 (E14/#1)	CP	0.06	0.83**
KO-21 (E14/#1)	CP		0.04
KO-22 (E14/#1)	MZ	0.63	
KO-23 (E14/#1)	MZ		1.33
KO-25 (E14/#1)	MZ		0.15
KO-26 (E14/#4)	MZ	0.18+0.07=0.25	
KO-27 (E14/#4)	MZ	0.02+0.10+0.20=0.32	
KO-33 (E14/#3)	MZ	0.45	
Cell not numbered (E14/#1)	MZ		0.38**
Cell not numbered (E14/#4)	MZ	0.21	
Cell not numbered (E14/#4)	MZ	0.20	
Cell not numbered (E14/#3)	MZ	0.36	
Cell not numbered (E14/#3)	MZ	0.30	
N (of them blocked by adjacent cells)		16 (N/A)	13 (4)
MEAN ± SEM		0.21 ±0.01	0.62 ±0.04
**CP-55940 doze 0.03 mg/kg body weight**			
CP4 (E13/#2)	CP	0.05+0.08=0.13	
Cell not numbered (E13/#2)	CP	0.26	
CP10 (E13/#2)	MZ		0.18
CP11 (E13/#2)	MZ		0.03**+0.05**=0.08
CP12 (E13/#2)	MZ	0.46	0.24
CP14 (E13/#1)	MZ	0.47	

Cell not numbered (E13/#4)	MZ		0.19
N (of them blocked by adjacent cells)		5 (N/A)	5 (2)
MEAN ± SEM		0.26 ±0.04	0.14 ±0.02
**CP-55940 doze 0.12 mg/kg body weight**			
CP27 (E12/#1)	IZ		0.20
CP28 (E12/#1)	IZ	0.15	
Cell not numbered (E12/#3)	CP	0.24	
Cell not numbered (E12/#3)	CP		1.63**
CP40 (E12/#4)	MZ		0.54**
CP41 (E12/#4)	MZ	0.43	
CP42 (E12/#4)	MZ		0.88**
CP44 (E12/#4)	MZ		0.24
CP47 (E12/#1)	MZ	0.36+0.07=0.43	
CP48 (E12/#1)	MZ	1.03+0.61=1.64	
CP49 (E12/#1)	MZ	0.06+0.03=0.09	
Cell not numbered (E12/#1)	MZ	0.93	
Cell not numbered (E12/#1)	MZ	0.13	
Cell not numbered (E12/#1)	MZ		0.18**
Cell not numbered (E12/#1)	MZ	0.09	
Cell not numbered (E12/#1)	MZ	0.13	
Cell not numbered (E12/#1)	MZ	0.05	
Cell not numbered (E12/#1)	MZ		0.37**
Cell not numbered (E12/#2)	MZ		0.54
Cell not numbered (E12/#3)	MZ	0.10	
Cell not numbered (E12/#3)	MZ		0.82**
Cell not numbered (E12/#3)	MZ		0.21**
Cell not numbered (E12/#3)	MZ	0.21	
Cell not numbered (E12/#3)	MZ		0.03**
Cell not numbered (E12/#3)	MZ	0.18	
Cell not numbered (E12/#3)	MZ		0.05
Cell not numbered (E12/#4)	MZ		0.26**
Cell not numbered (E12/#4)	MZ		0.43**
Cell not numbered (E12/#4)	MZ		0.19**
N (of them blocked by adjacent cells)		17 (N/A)	15 (11)
MEAN ± SEM		0.28 ±0.02	0.44 ±0.03
**WIN 55,212-2 doze 0.5 mg/kg body weight**			
WIN15 (E13/#2)	MZ		0.19
WIN17 (E13/#2)	MZ	0.07	0.10
WIN18 (E13/#2)	MZ	0.06+0.08=0.14	0.14**
Cell not numbered (E13/#2)	MZ	0.30	
N (of them blocked by adjacent cells)		4 (N/A)	3 (1)
MEAN ± SEM		0.13 ±0.03	0.14 ±0.02
**WIN 55,212-2 doze 2.0 mg/kg body weight**			
WIN31 (E13/#1)	IZ		0.18
Cell not numbered (E13/#1)	IZ		1.63
WIN37 (E13/#2)	CP		0.13

WIN39 (E13/#2)	CP	0.94	0.32
WIN40 (E13/#2)	CP	0.10	0.07
Cell not numbered (E13/#2)	CP	0.54	
Cell not numbered (E13/#2)	CP	0.04	
Cell not numbered (E13/#2)	CP	0.70	
Cell not numbered (E13/#2)	CP	0.93	
Cell not numbered (E13/#2)	CP		0.67**
WIN41 (E13/#3)	MZ		0.46
WIN43 (E13/#2)	MZ	0.16	0.15**
WIN45 (E13/#2)	MZ		0.92
WIN47 (E13/#4)	MZ	0.14+0.11+0.05=0.30	0.75+0.15=0.90
WIN48 (E13/#4)	MZ	0.14+0.06=0.20	
Cell not numbered (E13/#2)	MZ		0.13**
Cell not numbered (E13/#2)	MZ	0.16	
Cell not numbered (E13/#2)	MZ	0.11	
Cell not numbered (E13/#2)	MZ	0.39	
Cell not numbered (E13/#2)	MZ	0.24	
Cell not numbered (E13/#2)	MZ		0.69
Cell not numbered (E13/#2)	MZ	0.09	
Cell not numbered (E13/#2)	MZ		0.15**
Cell not numbered (E13/#2)	MZ		0.09
Cell not numbered (E13/#3)	MZ		0.20+0.18+0.16=0.54
Cell not numbered (E13/#3)	MZ	0.32	
Cell not numbered (E13/#4)	MZ		0.35**
Cell not numbered (E13/#4)	MZ		0.58**
Cell not numbered (E13/#4)	MZ		0.44**
Cell not numbered (E13/#4)	MZ	0.36	
N (of them blocked by adjacent cells)		19 (N/A)	21 (7)
MEAN ± SEM		0.29 ±0.02	0.40 ±0.02
Total N (of them blocked by adjacent cells)		65 (N/A)	57 (25)

† - Not numbered cells had incompletely reconstructed nuclei and were excluded from estimations of the frequency of hernias and morphometry of mitochondria.

* - Sum of volumes of several hernias in one cell.

** - Plasma membrane rupture is blocked by adjacent cells.

N is the number of 3D reconstructed hernias.

Abbreviations: IZ, intermediate zone; CP, cortical plate; MZ, marginal zone; N/A, not applicable.

Table S2.

Morphological characteristics of the 3D reconstructed neocortical neurons from the control wild type mouse embryos.

Cell code (embryo age/#)	Cell body location	Type of the cell body morphology	Number of emitted processes	Position of mother centriole	Volume of the nucleus, μm^3	Number of anti-CB_1R depositions		Number of the hernias	Total volume of the hernias, μm^3	Mitochondria		
						Single *	Globule **			N	Mean length ±SD, µm	Mean diameter ±SD, µm
M8 (E16/#2)	IZ	Vertical	2	Cytoplasm	99.3	3	0	0	0.00	40	1.15±0.85	0.30±0.03
M9 (E16/#2)	IZ	Vertical	2	Cytoplasm	79.9	3	0	0	0.00	40	1.07±0.63	0.29±0.04
M10 (E16/#2)	IZ	Vertical	2	Cytoplasm	100.9	2	0	0	0.00	40	1.00±0.62	0.31±0.04
M11 (E16/#2)	IZ	Vertical	2	Cytoplasm	142.4	4	2	0	0.00	40	1.46±0.96	0.32±0.04
M12 (E16/#3)	IZ	Vertical	1	Cell membrane	81.4	0	0	0	0.00	40	1.31±0.90	0.35±0.04
M13 (E16/#3)	IZ	Vertical	1	Cell membrane	78.0	0	0	0	0.00	40	1.77±1.61	0.32±0.04
M14 (E16/#3)	IZ	Multipolar	3	Cell membrane	114.7	13	1	0	0.00	40	1.23±0.99	0.33±0.05
M15 (E16/#3)	IZ	Horizontal	2	Cell membrane	138.8	13	9	0	0.00	40	1.01±0.49	0.35±0.05
M16 (E16/#3)	CP	Vertical	2	Cell membrane	101.3	0	0	0	0.00	40	1.27±0.75	0.28±0.03
M17 (E16/#3)	CP	Vertical	2	Cytoplasm	106.6	0	0	0	0.00	40	1.29±0.93	0.29±0.03
M18 (E16/#3)	CP	Vertical	2	Not found	105.4	0	0	0	0.00	40	1.46±0.96	0.30±0.03
M19 (E16/#3)	CP	Vertical	2	Not found	109.3	0	0	0	0.00	32	1.28±0.98	0.29±0.03
M20 (E16/#3)	CP	Vertical	2	Not found	112.4	0	0	0	0.00	39	1.06±0.82	0.33±0.04
M21 (E16/#3)	CP	Vertical	2	Not found	110.4	0	0	0	0.00	26	1.67±1.44	0.30±0.05
M22 (E16/#3)	CP	Vertical	2	Cytoplasm	95.8	0	0	0	0.00	40	1.24±0.76	0.30±0.04
M23 (E16/#3)	CP	Vertical	3	Cytoplasm	85.4	0	0	0	0.00	40	1.23±0.73	0.27±0.03
M24 (E16/#3)	CP	Vertical	4	Cell membrane	114.6	2	1	0	0.00	40	1.13±0.75	0.29±0.03
M25 (E16/#3)	CP	Vertical	3	Cytoplasm	75.6	0	0	0	0.00	40	0.96±0.59	0.27±0.03
M26 (E16/#4)	CP	Vertical	3	Cell membrane	125.7	8	1	0	0.00	40	1.06±0.69	0.30±0.03
M27 (E16/#4)	CP	Vertical	2	Cytoplasm	126.6	8	2	0	0.00	40	1.16±0.71	0.32±0.04
M28 (E16/#4)	CP	Vertical	2	Not found	70.6	0	0	0	0.00	23	0.93±0.66	0.25±0.03
M29 (E16/#4)	CP	Vertical	2	Cilial vesicle	107.9	0	1	0	0.00	40	1.26±0.78	0.29±0.03
M30 (E16/#4)	CP	Vertical	2	Cilial vesicle	95.5	2	1	0	0.00	40	1.07±0.82	0.27±0.03
M31 (E16/#4)	MZ	Horizontal	2	Cytoplasm	77.0	3	0	0	0.00	40	1.08±0.62	0.30±0.03
M32 (E16/#3)	MZ	Horizontal	2	Cytoplasm	143.9	32	24	1	0.07	40	1.69±1.14	0.26±0.04
M33 (E16/#3)	MZ	Horizontal	2	Cytoplasm	147.1	39	23	2	1.08	40	1.68±0.75	0.30±0.04
M34 (E16/#3)	MZ	Horizontal	2	Cytoplasm	101.9	5	1	0	0.00	40	1.22±0.62	0.28±0.06
M35 (E12/#1)	MZ	Horizontal	2	Cytoplasm	102.0	19	1	0	0.00	40	1.03±0.66	0.33±0.02
M36 (E12/#1)	MZ	Horizontal	2	Cilial vesicle	100.8	30	2	0	0.00	40	1.86±1.71	0.31±0.03
M37 (E12/#1)	MZ	Horizontal	2	Cilial vesicle	113.2	33	5	0	0.00	40	1.80±1.51	0.34±0.04
M38 (E12/#1)	MZ	Vertical	2	Procilium	125.6	51	5	0	0.00	40	0.83±0.42	0.35±0.03

M39 (E12/#1)	MZ	Horizontal	5	Cell membrane	105.6	54	4	0	0.00	40	1.17±0.75	0.37±0.06
M40 (E13/#1)	MZ	Horizontal	3	Procilium	112.8	2	2	1	0.05	32	1.04±1.14	0.32±0.05
M41 (E13/#1)	MZ	Horizontal	5	Cytoplasm	112.8	5	9	0	0.00	22	1.17±0.59	0.27±0.04
M42 (E13/#1)	MZ	Horizontal	7	Cytoplasm	110.0	7	8	0	0.00	21	1.35±0.74	0.28±0.04
M43 (E13/#1)	MZ	Horizontal	8	Cytoplasm	121.0	18	15	0	0.00	40	1.24±0.78	0.26±0.03
M44 (E13/#1)	MZ	Horizontal	9	Cytoplasm	144.4	13	20	0	0.00	40	1.37±0.81	0.27±0.05
M45 (E14/#1)	MZ	Horizontal	2	Cell membrane	80.0	0	0	0	0.00	40	1.01±0.79	0.38±0.07
M46 (E14/#1)	MZ	Horizontal	3	Procilium	148.4	18	16	0	0.00	40	1.22±1.19	0.35±0.06
M47 (E14/#1)	MZ	Horizontal	3	Cell membrane	86.8	0	0	0	0.00	40	0.74±0.41	0.41±0.11
M48 (E14/#1)	MZ	Horizontal	4	Cytoplasm	87.6	0	0	0	0.00	40	0.91±0.57	0.35±0.08
M49 (E14/#1)	MZ	Horizontal	3	Cell membrane	76.8	0	0	0	0.00	28	0.80±0.60	0.34±0.06

Abbreviations: IZ, intermediate zone; CP, cortical plate; MZ, marginal zone.

* - Single – small depositions of DAB-Ni in cytoplasm.

** - Globule – conglomerates of DAB-Ni depositions around intracellular vesicles.

Morphological characteristics of the cells M8-M34 were published in our previous article [S1] (CC BY http://creativecommons.org/licenses/by/4.0).

Table S3.

Morphological characteristics of the 3D reconstructed neocortical neurons from $CB_1R^{-/-}$ mouse embryos.

Cell code (embryo age/#)	Cell body location	Type of the cell body morphology	Number of emitted processes	Position of mother centriole	Volume of the nucleus, μm³	Number of the hernias	Total volume of the hernias, μm³	Mitochondria		
								N	Mean length ±SD, μm	Mean diameter ±SD, μm
KO1 (E14/#1)	IZ	Multipolar	4	Cytoplasm	68.0	1	0.88	24	0.63±0.35	0.34±0.06
KO2 (E14/#1)	IZ	Multipolar	8	Cilial vesicle	71.6	1	0.51	38	0.53±0.21	0.34±0.05
KO3 (E14/#1)	IZ	Vertical	2	Cell membrane †	77.6	0	0.00	42	0.87±0.97	0.34±0.04
KO4 (E14/#1)	IZ	Multipolar	3	Cilial vesicle	66.0	0	0.00	46	0.75±0.65	0.35±0.04
KO5 (E14/#1)	IZ	Multipolar	7	Cytoplasm	59.6	1	034	2	N/A	N/A
KO6 (E14/#1)	IZ	Multipolar	8	Cilial vesicle	70.8	1	0.68	15	0.78±0.54	0.33±0.04
KO7 (E14/#1)	IZ	Vertical	1	Not found	62.8	0	0.00	5	N/A	N/A
KO8 (E14/#2)	IZ	Multipolar	4	Not found	82.0	0	0.00	16	0.84±0.51	0.32±0.06
KO9 (E14/#2)	IZ	Vertical	2	Cilial vesicle	97.6	0	0.00	106	0.85±0.79	0.35±0.07
KO10 (E14/#2)	IZ	Vertical	2	Cytoplasm	96.8	2	0.23	71	0.89±0.64	0.36±0.07
KO11 (E14/#2)	IZ	Multipolar	8	Cilial vesicle	67.6	1	0.13	59	0.85±0.65	0.29±0.05
KO12 (E14/#2)	IZ	Multipolar	3	Cytoplasm	84.0	0	0.00	50	1.43±1.01	0.36±0.04
KO13 (E14/#3)	CP	Vertical	1	Not found	117.2	0	0.00	15	1.11±0.52	0.44±0.12
KO14 (E14/#3)	CP	Vertical	2	Not found	119.6	0	0.00	18	1.51±0.94	0.44±0.08
KO15 (E14/#3)	CP	Vertical	2	Not found	90.4	0	0.00	1	N/A	N/A
KO16 (E14/#3)	CP	Vertical	2	Not found	112.8	0	0.00	15	1.20±0.65	0.46±0.09
KO17 (E14/#1)	CP	Vertical	2	Cytoplasm	125.2	0	0.00	40	1.41±1.02	0.37±0.08
KO18 (E14/#1)	CP	Vertical	2	Procilium	94.8	0	0.00	40	1.62±1.01	0.34±0.08
KO19 (E14/#1)	CP	Vertical	3	Cytoplasm	93.2	1	0.07	24	0.66±0.33	0.39±0.09
KO20 (E14/#1)	CP	Vertical	2	Cytoplasm	111.6	2	0.89	20	0.96±0.57	0.39±0.09
KO21 (E14/#1)	CP	Vertical	2	Cilial vesicle	86.4	1	0.04	28	1.16±0.82	0.38±0.08
KO22 (E14/#1)	MZ	Horizontal	4	Cytoplasm	72.5	1	0.63	114	0.61±0.28	0.31±0.06
KO23 (E14/#1)	MZ	Horizontal	3	Not found	82.1	1	1.33	40	0.56±0.32	0.35±0.05
KO24 (E14/#1)	MZ	Horizontal	7	Cytoplasm	107.6	0	0.00	40	0.84±0.64	0.39±0.06
KO25 (E14/#1)	MZ	Horizontal	2	Not found	78.8	1	0.15	40	0.46±0.13	0.30±0.05
KO26 (E14/#4)	MZ	Horizontal	4	Cytoplasm	140.8	2	0.25	40	0.91±0.56	0.34±0.09
KO27 (E14/#4)	MZ	Horizontal	3	Cytoplasm †	129.0	3	0.32	40	1.30±0.85	0.35±0.09
KO28 (E14/#4)	MZ	Horizontal	1	Cytoplasm	85.2	0	0.00	40	0.74±0.43	0.31±0.04

KO29 (E14/#4)	MZ	Horizontal	2	Cytoplasm	84.8	0	0.00	40	0.64±0.30	0.34±0.05
KO30 (E14/#3)	MZ	Horizontal	4	Not found	68.8	0	0.00	40	0.69±0.32	0.40±0.09
KO31 (E14/#3)	MZ	Horizontal	3	Not found	67.6	0	0.00	40	0.67±0.38	0.38±0.10
KO32 (E14/#3)	MZ	Horizontal	11	Cytoplasm	101.8	0	0.00	33	1.19±0.94	0.32±0.06
KO33 (E14/#3)	MZ	Horizontal	2	Not found	84.0	1	0.45	36	0.53±0.16	0.33±0.04
KO34 (E14/#3)	MZ	Horizontal	4	Cytoplasm	118.3	0	0.00	40	0.97±0.65	0.34±0.12

† - Centrosome is duplicated during early prophase.

Abbreviations: IZ, intermediate zone; CP, cortical plate; MZ, marginal zone.

Morphological characteristics of the cells KO1-KO16 were published in our previous article [S1] (CC BY http://creativecommons.org/licenses/by/4.0).

Table S4.

Morphological characteristics of the 3D reconstructed neocortical neurons from wild type mouse embryos exposed to CB_1R agonist CP-55940.

Cell code (embryo age/#)	Cell body location	Type of the cell body morphology	Number of emitted processes	Position of mother centriole	Volume of the nucleus, μm^3	Number of anti-CB_1R depositions		Number of the hernias	Total volume of the hernias, μm^3	Mitochondria		
						Single *	Globule **			N	Mean length ±SD, μm	Mean diameter ±SD, μm
**Doze 0.03 mg/kg body weight**												
CP1 (E13/#1)	IZ	Vertical	5	Cilial vesicle	119.6	4	6	0	0.00	40	1.05±0.53	0.35±0.05
CP2 (E13/#1)	IZ	Horizontal	2	Procilium	109.2	1	2	0	0.00	40	1.44±0.89	0.32±0.03
CP3 (E13/#1)	IZ	Horizontal	3	Cytoplasm	108.0	2	4	0	0.00	40	1.53±1.29	0.31±0.03
CP4 (E13/#2)	CP	Vertical	3	Not found	143.6	0	0	2	0.13	40	1.33±0.91	0.39±0.05
CP5 (E13/#2)	CP	Vertical	3	Not found	78.8	0	0	0	0.00	40	0.75±0.44	0.36±0.03
CP6 (E13/#3)	CP	Vertical	2	Procilium	107.2	0	0	0	0.00	32	0.94±0.55	0.28±0.04
CP7 (E13/#3)	CP	Horizontal	5	Cilium	67.6	0	0	0	0.00	32	0.77±0.68	0.27±0.04
CP8 (E13/#3)	CP	Horizontal	3	Procilium	65.6	0	0	0	0.00	21	0.83±0.49	0.30±0.05
CP9 (E13/#2)	MZ	Horizontal	6	Cytoplasm	189.5	21	11	0	0.00	40	1.87±1.40	0.30±0.05
CP10 (E13/#2)	MZ	Horizontal	5	Not found	124.1	0	1	1	0.18	28	1.24±0.83	0.39±0.05
CP11 (E13/#2)	MZ	Horizontal	9	Cilial vesicle	100.2	18	5	2	0.08	40	1.55±1.59	0.36±0.04
CP12 (E13/#2)	MZ	Horizontal	7	Cilial vesicle	102.1	21	11	2	0.70	40	0.92±0.68	0.33±0.04
CP13 (E13/#1)	MZ	Horizontal	5	Cilium	90.0	0	0	0	0.00	40	1.14±0.52	0.33±0.03
CP14 (E13/#1)	MZ	Horizontal	4	Not found	89.6	0	0	1	0.47	14	1.66±1.08	0.34±0.04
CP15 (E13/#1)	MZ	Horizontal	8	Cytoplasm	91.6	0	0	0	0.00	35	1.19±0.68	0.37±0.04
CP16 (E13/#1)	MZ	Horizontal	6	Cytoplasm	140.2	2	7	0	0.00	40	1.34±0.87	0.28±0.04
CP17 (E13/#4)	MZ	Horizontal	7	Cytoplasm	112.4	19	14	0	0.00	40	1.33±0.08	0.34±0.03
CP18 (E13/#4)	MZ	Vertical	4	Procilium	73.6	0	0	0	0.00	18	0.78±0.35	0.35±0.04
CP19 (E13/#4)	MZ	Horizontal	5	Cilial vesicle	102.8	6	11	0	0.00	40	1.37±1.08	0.32±0.04
CP20 (E13/#4)	MZ	Horizontal	4	Cytoplasm	114.0	12	16	0	0.00	40	1.06±0.71	0.33±0.04
CP21 (E13/#4)	MZ	Horizontal	4	Procilium	80.4	0	0	0	0.00	40	1.00±0.66	0.35±0.05
CP22 (E13/#4)	MZ	Horizontal	3	Cytoplasm	96.0	0	0	0	0.00	40	0.82±0.67	0.27±0.04
CP23 (E13/#4)	MZ	Horizontal	7	Cytoplasm	98.8	1	2	0	0.00	9	N/A	N/A
**Doze 0.12 mg/kg body weight**												
CP24 (E12/#1)	IZ	Vertical	4	Procilium	96.2	0	0	0	0.00	40	1.14±1.10	0.33±0.05
CP25 (E12/#1)	IZ	Vertical	2	Not found	102.4	0	0	0	0.00	40	1.26±1.03	0.32±0.03
CP26 (E12/#1)	IZ	Vertical	6	Procilium	87.6	0	0	0	0.00	40	1.22±0.91	0.35±0.03
CP27 (E12/#1)	IZ	Horizontal	4	Procilium	133.4	26	14	1	0.20	40	0.98±0.81	0.35±0.05
CP28 (E12/#1)	IZ	Horizontal	4	Cell membrane	96.8	15	0	1	0.15	40	1.08±0.74	0.39±0.04

CP29 (E12/#1)	IZ	Horizontal	1	Cilial vesicle	96.6	3	0	0	0.00	40	0.76±0.46	0.37±0.04
CP30 (E12/#2)	CP	Vertical	2	Not found	161.2	11	0	0	0.00	27	1.44±0.93	0.40±0.06
CP31 (E12/#2)	CP	Vertical	2	Not found	203.2	4	0	0	0.00	7	N/A	N/A
CP32 (E12/#2)	CP	Vertical	4	Not found	178.4	6	0	0	0.00	30	1.55±1.05	0.42±0.05
CP33 (E12/#2)	CP	Vertical	2	Not found	150.2	8	0	0	0.00	19	1.43±1.02	0.42±0.06
CP34 (E12/#2)	CP	Vertical	2	Not found	181.6	0	0	0	0.00	24	1.34±0.76	0.42±0.08
CP35 (E12/#2)	MZ	Horizontal	2	Procilium	139.6	63	10	0	0.00	40	1.25±0.96	0.32±0.04
CP36 (E12/#2)	MZ	Horizontal	3	Cytoplasm	148.4	69	6	0	0.00	40	1.86±1.54	0.32±0.03
CP37 (E12/#3)	MZ	Horizontal	5	Procilium	119.6	54	12	0	0.00	40	1.69±1.18	0.27±0.02
CP38 (E12/#3)	MZ	Horizontal	7	Procilium	88.6	39	1	0	0.00	29	1.23±0.61	0.35±0.04
CP39 (E12/#4)	MZ	Multipolar	4	Cytoplasm	133.2	35	7	0	0.00	40	1.37±0.97	0.30±0.04
CP40 (E12/#4)	MZ	Horizontal	2	Cilial vesicle	135.3	36	12	1	0.54	40	1.33±1.17	0.31±0.04
CP41 (E12/#4)	MZ	Horizontal	12	Not found	139.6	44	3	1	0.10	40	1.32±0.78	0.29±0.03
CP42 (E12/#4)	MZ	Horizontal	2	Cilial vesicle	167.4	76	16	1	0.88	40	1.29±0.64	0.31±0.03
CP43 (E12/#4)	MZ	Horizontal	5	Not found	105.2	0	0	0	0.00	32	1.32±1.15	0.33±0.04
CP44 (E12/#4)	MZ	Horizontal	2	Cilium	104.4	12	0	1	0.24	40	0.89±0.50	0.32±0.05
CP45 (E12/#4)	MZ	Multipolar	11	Cilium	82.0	11	0	0	0.00	40	1.68±1.33	0.33±0.04
CP46 (E12/#1)	MZ	Horizontal	2	Cilial vesicle	105.6	19	6	0	0.00	40	1.30±0.89	0.34±0.05
CP47 (E12/#1)	MZ	Horizontal	2	Not found	117.2	30	4	2	0.43	40	0.69±0.40	0.38±0.05
CP48 (E12/#1)	MZ	Horizontal	5	Cilial vesicle	148.1	81	21	2	1.64	40	1.08±0.80	0.36±0.07
CP49 (E12/#1)	MZ	Horizontal	2	Cilial vesicle	111.4	32	2	2	0.09	40	1.28±1.10	0.37±0.04

Abbreviations: IZ, intermediate zone; CP, cortical plate; MZ, marginal zone.

* - Single – small depositions of DAB-Ni in cytoplasm.

** - Globule – conglomerates of DAB-Ni depositions around intracellular vesicles.

Table S5.

Morphological characteristics of the 3D reconstructed neocortical neurons from wild type mouse embryos exposed to CB_1R agonist WIN 55,212-2.

Cell code (embryo age/#)	Cell body location	Cell body morphology	Number of emitted processes	Position of mother centriole	Volume of the nucleus, μm^3	Number of anti-CB_1R depositions		Number of the hernias	Total volume of the hernias, μm^3	Mitochondria		
						Single *	Globule **			N	Mean Length ±SD, μm	Mean Diameter ±SD, μm
**Doze 0.5 mg/kg body weight**												
WIN1 (E16/#1)	IZ	Vertical	2	Cytoplasm	115.2	0	0	0	0.00	40	1.06±0.81	0.31±0.04
WIN2 (E16/#1)	IZ	Vertical	2	Procilium	130.8	0	0	0	0.00	40	1.42±0.86	0.31±0.04
WIN3 (E16/#1)	IZ	Horizontal	2	Cytoplasm	128.0	0	0	0	0.00	40	1.55±1.24	0.34±0.05
WIN4 (E16/#1)	IZ	Horizontal	3	Cytoplasm	113.6	0	0	0	0.00	40	0.98±0.82	0.37±0.05
WIN5 (E16/#1)	IZ	Horizontal	2	Procilium	122.8	0	0	0	0.00	28	0.83±0.85	0.33±0.05
WIN6 (E13/#1)	CP	Vertical	2	Cytoplasm	118.2	0	1	0	0.00	27	1.04±0.59	0.32±0.06
WIN7 (E13/#1)	CP	Vertical	2	Not found	127.2	6	1	0	0.00	23	1.32±1.42	0.33±0.04
WIN8 (E13/#1)	CP	Vertical	2	Procilium	108.5	3	0	0	0.00	40	0.91±0.61	0.33±0.05
WIN9 (E13/#1)	CP	Vertical	2	Cytoplasm	114.0	3	1	0	0.00	20	1.21±0.71	0.39±0.07
WIN10 (E13/#1)	CP	Vertical	2	Cytoplasm	94.8	0	0	0	0.00	22	1.37±0.99	0.36±0.08
WIN11 (E13/#1)	CP	Vertical	2	Cytoplasm	108.6	6	0	0	0.00	2	N/A	N/A
WIN12 (E12/#1)	MZ	Horizontal	3	Cell membrane	104.0	2	4	0	0.00	40	1.29±0.80	0.31±0.03
WIN13 (E12/#1)	MZ	Horizontal	2	Not found	126.2	0	3	0	0.00	40	1.02±0.72	0.31±0.03
WIN14 (E13/#2)	MZ	Horizontal	10	Cilium	86.4	0	0	0	0.00	40	1.02±0.60	0.38±0.06
WIN15 (E13/#2)	MZ	Horizontal	5	Cilium	89.2	8	0	1	0.19	40	1.11±0.60	0.37±0.05
WIN16 (E13/#2)	MZ	Horizontal	12	Procilium	100.2	32	10	0	0.00	40	2.01±1.91	0.33±0.04
WIN17 (E13/#2)	MZ	Horizontal	7	Cytoplasm	113.6	22	9	2	0.17	40	1.68±1.53	0.37±0.04
WIN18 (E13/#2)	MZ	Vertical	3	Not found	90.4	0	0	3	0.28	2	N/A	N/A
WIN19 (E13/#2)	MZ	Vertical	2	Not found	89.6	0	0	0	0.00	20	1.15±0.57	0.38±0.04
WIN20 (E13/#3)	MZ	Vertical	4	Cilial vesicle	125.5	6	9	0	0.00	40	1.27±0.72	0.37±0.05
WIN21 (E13/#3)	MZ	Horizontal	5	Cytoplasm	87.2	0	0	0	0.00	40	0.79±0.56	0.29±0.05
WIN22 (E13/#3)	MZ	Horizontal	3	Cell membrane	136.8	16	14	0	0.00	40	1.08±0.64	0.32±0.07
WIN23 (E13/#3)	MZ	Horizontal	2	Cilial vesicle	141.2	20	11	0	0.00	40	0.62±0.32	0.35±0.08
WIN24 (E16/#1)	MZ	Horizontal	2	Not found	137.6	0	0	0	0.00	36	0.59±0.26	0.37±0.06
WIN25 (E16/#1)	MZ	Horizontal	2	Cytoplasm	114.4	0	0	0	0.00	40	0.79±0.63	0.32±0.04
WIN26 (E16/#1)	MZ	Horizontal	2	Cell membrane	110.8	0	0	0	0.00	40	0.77±0.48	0.32±0.03
WIN27 (E16/#1)	MZ	Vertical	2	Cytoplasm	145.4	13	13	0	0.00	40	0.89±0.56	0.32±0.03
WIN28 (E16/#1)	MZ	Vertical	2	Cytoplasm	137.0	20	1	0	0.00	40	1.06±0.63	0.30±0.03
WIN29 (E16/#1)	MZ	Horizontal	2	Not found	109.6	0	0	0	0.00	40	1.11±1.07	0.27±0.02
WIN30 (E16/#1)	MZ	Horizontal	2	Cytoplasm	144.0	17	3	0	0.00	40	0.71±0.40	0.33±0.03

Doze 2.0 mg/kg body weight												
WIN31 (E13/#1)	IZ	Multipolar	5	Not found	125.2	0	0	1	0.18	22	1.32±089	0.38±0.03
WIN32 (E13/#1)	IZ	Horizontal	4	Cytoplasm	90.0	0	0	0	0.00	40	0.98±0.69	0.33±0.03
WIN33 (E13/#1)	IZ	Multipolar	9	Procilium	77.2	2	1	0	0.00	40	0.87±0.54	0.36±0.04
WIN34 (E13/#1)	IZ	Multipolar	4	Procilium	94.8	6	10	0	0.00	40	1.33±0.92	0.35±0.04
WIN35 (E13/#1)	IZ	Horizontal	3	Procilium	75.2	0	0	0	0.00	40	1.25±1.06	0.33±0.04
WIN36 (E13/#1)	IZ	Horizontal	5	Cilium	80.7	0	0	0	0.00	40	1.00±0.69	0.37±0.04
WIN37 (E13/#2)	CP	Vertical	3	Not found	149.2	0	0	1	0.13	16	1.67±0.76	0.41±0.06
WIN38 (E13/#2)	CP	Vertical	2	Not found	136.4	0	0	0	0.00	14	1.45±1.34	0.42±0.06
WIN39 (E13/#2)	CP	Vertical	3	Not found	146.0	0	0	2	1.26	22	1.25±0.89	0.41±0.05
WIN40 (E13/#2)	CP	Vertical	2	Not found	136.0	0	0	2	0.17	16	1.60±1.12	0.42±0.05
WIN41 (E13/#3)	MZ	Horizontal	6	Not found	86.4	26	1	1	0.46	12	1.11±0.67	0.35±0.02
WIN42 (E13/#3)	MZ	Horizontal	7	Procilium	73.8	0	0	0	0.00	27	1.76±1.36	0.36±0.03
WIN43 (E13/#2)	MZ	Horizontal	7	Procilium	101.8	4	15	2	0.31	40	2.23±1.58	0.33±0.05
WIN44 (E13/#2)	MZ	Horizontal	6	Cilial vesicle	79.6	0	0	0	0.00	40	0.94±0.63	0.36±0.04
WIN45 (E13/#2)	MZ	Horizontal	5	Cytoplasm	91.2	3	8	1	0.92	40	1.33±0.84	0.35±0.04
WIN46 (E13/#2)	MZ	Horizontal	11	Not found	77.6	0	0	0	0.00	15	1.16±0.82	0.36±0.05
WIN47 (E13/#4)	MZ	Horizontal	4	Cilium	118.8	12	11	5	1.20	40	1.14±0.84	0.33±0.03
WIN48 (E13/#4)	MZ	Horizontal	5	Cytoplasm	104.0	0	4	2	0.20	40	1.35±0.77	0.37±0.04
WIN49 (E13/#4)	MZ	Horizontal	2	Not found	100.0	1	5	0	0.00	35	1.18±0.82	0.36±0.03
WIN50 (E13/#4)	MZ	Horizontal	6	Cilium	110.0	0	7	0	0.00	40	0.88±0.55	0.33±0.04
WIN51 (E13/#4)	MZ	Horizontal	3	Cilium	128.0	2	14	0	0.00	40	1.56±0.95	0.33±0.03
WIN52 (E13/#4)	MZ	Horizontal	5	Cytoplasm	94.0	1	3	0	0.00	40	1.33±0.84	0.37±0.04

Abbreviations: IZ, intermediate zone; CP, cortical plate; MZ, marginal zone.

* - Single – small depositions of DAB-Ni in cytoplasm.

** - Globule – conglomerates of DAB-Ni depositions around intracellular vesicles.

Table S6.

Degree of CB_1R accumulation in the cell bodies of herniated cells.

Cell code (embryo age/#)	Cell body location	Degree of CB_1R expression	
		Cells with NE ruptures	Cells with PNH
M32 (E16/#3)	MZ	High (32; 24) *	
M33 (E16/#3)	MZ	High (39; 23)	
M40 (E13/#1)	MZ	Middle (2; 2)	
CP4 (E13/#2)	CP	Absent (0; 0)	
CP10 (E13/#2)	MZ		Low (0; 1)
CP11 (E13/#2)	MZ		High (18; 5)
CP12 (E13/#2)	MZ		High (21; 11)
CP14 (E13/#1)	MZ	Absent (0; 0)	
CP27 (E12/#1)	IZ		High (26; 14)
CP28 (E12/#1)	IZ	Low (12; 0)	
CP40 (E12/#4)	MZ		High (36; 12)
CP41 (E12/#4)	MZ	High (44; 3)	
CP42 (E12/#4)	MZ		High (76; 16)
CP44 (E12/#4)	MZ		Low (12; 0)
CP47 (E12/#1)	MZ	High (30; 4)	
CP48 (E12/#1)	MZ	High (81; 21)	
CP49 (E12/#1)	MZ	Middle (32; 2)	
WIN15 (E13/#2)	MZ		Low (8; 0)
WIN17 (E13/#2)	MZ		High (22; 9)
WIN18 (E13/#2)	MZ		Absent (0; 0)
WIN31 (E13/#1)	IZ		Absent (0; 0)
WIN37 (E13/#2)	CP		Absent (0; 0)
WIN39 (E13/#2)	CP		Absent (0; 0)
WIN40 (E13/#2)	CP		Absent (0; 0)
WIN41 (E13/#3)	MZ		Low (26; 1)
WIN43 (E13/#2)	MZ		High (4; 15)
WIN45 (E13/#2)	MZ		High (3; 8)
WIN47 (E13/#4)	MZ		High (12; 11)
WIN48 (E13/#4)	MZ	Middle (0; 4)	
Number of cells with high degree of CB_1R expression		5 (of them 5 from MZ; 0 from IZ and CP)	9 (of them 8 from MZ; 1 from IZ and CP)
Number of cells with middle degree of CB_1R expression		3 (of them 3 from MZ; 0 from IZ and CP)	0
Number of CB_1R-negative cells or cells with low degree of CB_1R expression		3 (of them 1 from MZ; 2 from IZ and CP)	9 (of them 5 from MZ; 4 from IZ and CP)

* In parentheses, the first value is the number of single points of anti-CB_1R depositions; the second value is the number of CB_1R-positive globules (**Tables S2, S4** and **S5**).

Abbreviations: CP, cortical plate; IZ, intermediate zone; MZ, marginal zone.

Supplemental reference

S1. Morozov, Y.M., Mackie, K., and Rakic, P. (2020). Cannabinoid Type 1 Receptor is Undetectable in Rodent and Primate Cerebral Neural Stem Cells but Participates in Radial Neuronal Migration. International journal of molecular sciences *21*. 10.3390/ijms21228657.
